# Supplementary material for: Telomere-to-telomere assemblies of 142 strains characterize the genome structural landscape in Saccharomyces cerevisiae
Source: Nat Genet. 2023 Jul 31;55(8):1390–9. doi: 10.1038/s41588-023-01459-y (PMC10412453; doi:10.1038/s41588-023-01459-y)
Supplement: Supplementary file 1 — Supplementary Notes 1–3, Supplementary Methods and Supplementary Figs. 1–15. [file 41588_2023_1459_MOESM1_ESM.pdf]

nature genetics

Article

https://doi.org/10.1038/s41588-023-01459-y

Telomere-to-telomere assemblies of 142 strains characterize the genome structural landscape in *Saccharomyces cerevisiae*

In the format provided by the authors and unedited

Supplementary Information

This file contains supplementary notes 1-3, methods and figures 1-15.

Supplementary Note1: Additional details on genome assemblies

The ScRAP includes 100 newly sequenced genomes, (ii) 18 re-assembled genomes[1] and (iii) 24 publically available assemblies[2–9] (Supp. Fig. 1a, Supp. Table 2). Overall, the ScRAP consists of 142 haploid or collapsed assemblies (one per strain), 55 haplotype-resolved assemblies comprising two phased assemblies per heterozygous diploid (21 strains) and one haplo-phased assembly per heterozygous polyploid (13 strains), totaling 197 nuclear genome assemblies (Supp. Fig. 1b, Table 1, Supp. Table 1). The number of contigs/scaffolds per nuclear genome assembly reveals high contiguity for all haploid/collapsed assemblies (Supp. Fig. 2a,b, Supp. Table 1). Additionally, 14 phased diploid assemblies of our newly sequenced genomes show an unprecedented level of contiguity with an average of 25 scaffolds per haplotype. Seven of them are completely assembled into a single telomere-to-telomere haplotype-phased scaffold per chromosome. Based on genome size, the newly sequenced genomes are the most complete, being on average 120 kb and 80 kb larger than the re-assembled and publically available genomes, respectively (Supp. Fig. 2C). The number of chromosomes fully assembled from telomere to telomere is also higher in the newly sequenced genomes as compared to the two other datasets (Supp. Fig. 2d, Supp. Table 1). Noticeably, 47/100 haploid/collapsed assemblies include terminal telomeric repeats at all 32 chromosome ends while only 3/24 public assemblies (12.5%) and none of the 18 re-assembled genomes have all 32 telomeres. The SGD reference genome (S288C) only contains 21 telomeres despite the dedicated effort to reconstruct them[10]. Phased assemblies also approach a telomere-to-telomere completion level with a maximum of 60/64, 76/96 and 101/128 telomeres respectively for the diploid, triploid and tetraploid assemblies. The estimation of the mean telomere length per genome also confirms that the 100 *newly sequenced genomes* are the most complete of all with an average size of 340 bp as compared to 215 bp and 93 bp for public and re-assembled genomes, respectively (Supp. Fig. 2e).

The ScRAP contains only 136 mitochondrial assemblies because (i) 3 strains ALH_1c,

ASB and ASG) are petite mutants devoid of mitochondrial DNA, (ii) 2 strains have highly incomplete mitochondrial assemblies probably due to low coverage (BDH and RM11) and (iii) the BY4742 genome assembly does not include a mitochondrial contig.

Supplementary Note 2: Additional details on ScRAP phylogenies

We built a phylogenetic tree based on the concatenated protein sequence alignment of 1,618 1:1 orthologs conserved across the ScRAP and 23 strains from other *Saccharomyces* species used as outgroups to root the tree (Fig. 1c). This ortholog-based tree is consistent with previous phylogenetic reconstructions and provides reliable phylogenetic structure to previously loose mosaic clusters[11] and to isolated branches. Sister haplotypes (HP1 and HP2) from haplotype-resolved diploid assemblies always grouped together in the tree and shared the same admixture profile (Fig. 1c and 1d). This did not result from the random assortment of homologous chromosomes within each phased assembly as single-chromosome reconstructions also showed that both haplotypes shared a common phylogenetic origin rather than originating from recent admixture. Additionally, a total of 1,581,350 high quality reference-based SNPs detected across the ScRAP were used to build an alternative phylogenetic tree. The two tree topologies are similar (Clustering Information Distance of 0.18), differing mainly at terminal nodes by local alternative branching (Supp. Fig. 3).

The genetic composition of the clinical isolate YJM454 (ABH) strain shows a mixed origin between the 'American' and the 'Mosaic beer/cider' clades (Fig. 1d) that explains its phylogenetic positioning instability between the orthologue and SNV-based trees (Supp. Fig. 3). The 'Alpechin' lineage branches as a sister taxon to the 'Wine European 2' clade within the larger population of wine European strains. However, the Alpechin clade has a unique genomic composition, different from that of the wine European strains as it carries abundant *S. paradoxus* introgressions[11,12]. We defined three new clades called 'Cider', 'Lab-related' and 'Spanish' that comprise isolates previously belonging to the mosaic regions. The existence of these new clades is further supported by their inferred genetic ancestry (Fig. 1d). We also merged the 'sake' and 'asian fermentation' clades into a single 'Asian fermentation' group as well as the 'North America oak' and the 'Ecuadorian' clades into a new 'American' clade. In these two cases, the groupings are consistent with shared geographical

origins and their genetic ancestry (Fig. 1b and d). The American clade contains a Chinese strain (BJ4) from the CHNVI/VII clade originally defined in ref[13]. The genetic relatedness between the American and China VI/VII strains support a recent shared history that likely postdates the main out-of-China event that founded the rest of the world population[11]. The two alternative scenarios of China VI/VII that recently migrated to the Americas or American strains that have re-entered China remain to be defined. One clade containing only two strains isolated from a grape berry in far east Russia (CDG) and from a beetle in Bulgaria (BBL) remains undefined but the strains present an admixed genomic composition comparable to that of the American strains (Fig. 1d). The root of the ortholog-based tree groups together the two most diverged Taiwanese I and the Chinese IX wild populations, which is consistent with the East Asian geographical origin of the species[11,13–15].

We also built a phylogenetic tree using the 36,459 SVs identified among the 142 strains and compared its topology to that of the SNV-based tree (Supp. Fig. 4). The two trees show a globally conserved organization (Clustering Information Distance of 0.44) with the most diverged isolates corresponding to the Chinese and Taiwanese strains and most clades, as defined from the ortholog tree (Fig. 1c), remaining individualized and recognizable. However, the relative position of the clades is poorly conserved between the two trees. For instance the two sisters clades, 10. Mexican distillery/agave and 09. French Guiana human, remain grouped together in both trees but their position switched from a more external node in the SNVs/INDELs tree to a more internal node in the SV tree, close to the 18. African palm wine clade. These discrepancies could be due to a less reliable phylogenetic reconstruction in the SV-based tree as indicated by lower confidence scores, only 56% of the nodes are supported by confidence values higher than 0.95 as compared to 93% in the SNV-based tree (Supp. Fig. 5). This is likely due to a much lower number of distinct events (4,809 SVs *vs* 1,581,350 SNPs) as well as low allele frequencies for SVs (91% with MAF<0.1). In some cases, the evolutionary signal brought by SV-based tree is meaningful. For instance, the BJ4 strain, which was isolated from China and previously located in a Chinese clade[11], now branches in a more internal region of the tree along other Chinese strains (Supp. Fig. 4) while it clusters within, or next to the American clade containing primarily north American oak samples in both the ortholog-based and SNV-based trees (Fig. 1c, Supp. Fig. 4). Estimation of individual genetic ancestries by

ADMIXTURE (version: 1.3.0)[16] using SVs as markers also confirm the phylogenetic clustering defined by the SV-tree. For example, the DBVPG1841 (BPG) strain shares the same ancestry as the CBS7964 (AEH) strain, supporting their clustering in the SV-based tree, while these two isolates are located in different clades in both the ortholog and SNP-based trees (Supp. Fig. 4).

Supplementary Note 3: Additional details on complex regions

Simple and complex aneuploidies

We found that aneuploidies are highly unstable given that several cases are different between the initial state described in the population survey of 1,011 isolates[11] and the chromosomal content in this study. We found 6 aneuploidies, in 4 strains, that were lost since the initial Illumina sequencing, either by the gain (5) or loss (1) of a chromosome. Two out of the 6 lost aneuploid chromosomes appear to be complex aneuploidies that were previously undetected. Conversely, we also found 7 cases where chromosomes became newly aneuploid since the initial survey, of which 6 occurred in the monosporic isolates. We also noted that 5 cases were not initially reported[11], although present and 6 have changed their aneuploidy status (5 gained an extra copy and 1 changed from +1*chr1 to -1*chr1). Notably 3/5 aneuploid chromosomes not reported previously, were complex aneuploidies. More globally, the 1,011 survey reported 343 simple aneuploid chromosomes in 200 strains[11]. Reanalysing the same dataset here, we found 120 additional cases where 35 of them (29%) consisting of complex aneuploidies, showing that they were prevalently undetected in the initial analysis.

The proportion of aneuploid chromosomes estimated to be complex in the larger dataset of 993 strains (84 from ref[17] and 909 from ref[11]) is between 10-18% (44/423 - 85/464). In terms of strains, 15-23% (36/248 - 59/248) of aneuploid strains are predicted to contain at least one complex aneuploidy (Supp. Table 9, Supp. Table 10). Lower boundaries of 10% of complex aneuploid chromosomes in 15% of the strains are conservative estimates as they only consider Centromere-Related (CR) events *i.e.* regions where the coverage deviation covers a centromere (for instance chrVIII in AGA in Fig. 3a). The upper boundaries of 18% of complex aneuploidies in 23% of aneuploid strains are less conservative estimates as they also include chromosomes with large

(>100 kb) Non-Centromere Related (NCRs) coverage deviations, if the same strain includes another CR event (for instance chrXVI in AGA in Fig. 3a). It is fair to use the less conservative estimate including NCRs because of the clear association between CRs and both more frequent and large NCRs (Supp. Fig. 6), and also because we characterized several examples of large translocations being at the root of complex aneuploidies (Fig. 3a). False positive NCRs would only correspond to the co-occurrence of both a large segmental duplication (> 100kb) and at least one simple aneuploid chromosome in the same genome and therefore should remain limited. Therefore, if a strain contains an aneuploid chromosome and a large NCR, the NCR chromosome can be considered also as an aneuploid-related chromosome.

The detailed chromosomal structure of the complex aneuploidy in the strain CBS457 (AIF) could not be fully resolved as in addition to a chrXVI_chrIV aneuploid translocation, another complex chrXI_chrXIV aneuploid translocation was identified but its fine structure remained elusive.

Complex aneuploidy is similar to previously reported cases of 'segmental aneuploidies' in which two copies of the left arm of chromosome 5 were fused around a single copy of the centromere in several azole resistant strains of *Candida albicans*[18]. However, in many cases segmental aneuploidies contrast with our definition of complex aneuploidies whereby a centromere must be contained within the copy-number modified region. For instance, in humans, this is the case for chromosome arm aneuploidies (CAAs) which occur frequently in cancer and even more frequently than whole chromosome aneuploidies in certain cancer types[19,20]. These CAAs have the potential to represent complex aneuploidies as we have described here. However, due to the complex repetitiveness of human centromere regions, only recently have these regions been reliably assembled[21] and therefore centromere complexity may have hidden these cases in other aneuploidy rich data such as human cancer. Several studies in *Saccharomyces* reported a significant negative correlation between chromosome size and the rate of simple aneuploidy[22], probably because the fitness cost of extra chromosomes is proportional to the total number of genes present in the excess chromosomes. In line with this, CAAs frequency in human cancers is inversely related to arm length[23]. On the other hand, we showed that larger chromosomes are more often involved in complex aneuploidies than the smaller ones. Therefore, we hypothesize that complex aneuploidy offers a

powerful adaptive route that would be inaccessible to simple aneuploidies by allowing to combine the benefits of increased copy number of selected genes from large chromosomes and reducing the cost of gene imbalance through either large deletions that encompass deleterious genes or translocations. In addition, they are widespread in the population (up to 18% of all aneuploidies could be complex), while they should be extremely rare as they are expected to occur at a rate corresponding to the product of the rates of aneuploidy and SV formation. Therefore, complex aneuploidies could possibly be adaptive and selected for in response to harsh environments, as was demonstrated for simple aneuploidies[24]. Further work is needed to characterize the molecular mechanisms by which complex aneuploidies are formed and to determine their specific adaptive potential.

The content of Y' elements widely varies across strains

There are on average 17 Y' elements per genome. The majority of chromosome ends are devoid of Y' elements (58% or 2,642 out of 4,560), while 36% (1,660) have a single element (Supp. Fig. 7a). We found two strains that are completely devoid of Y' elements, one from Ecuador, CLQCA_20-156 (CCC_1a), and one from Nigeria, PW5_b (ADE, Supp. Fig. 7b). Noticeably, the haploid YJM981_b clinical isolate (ADI) harbors an extremely high number of Y'-element[25], with 154 subtelomeric assembled copies (full-length + partial) as compared to a median of 16.5 elements in the *de novo* haploid/collapsed assemblies (Supp. Table 11). As a result, this strain has the largest genome of all with a genome size that is 920 kb larger than the median genome size (12.89 Mb *vs* 11.97 Mb, Extended Data Fig. 10a).

HGT regions constitute new telomeres

Region B originates from *Zygosaccharomyces parabailii* [11,26]. We detected region B in 28 strains (Extended Data Fig. 8). We assembled a complete copy (98 kb) and closely inspected its boundaries. First, we observed a local region of homology (75%) including several tracts between 10 and 20 bp of complete identity near the insertion site (Supp. Fig. 8a). Such homology is highly unexpected given the extreme sequence divergence of the two species (>30%) and suggests that HGTs could possibly be driven by sequence homology, as for introgressions, their insertion sites might be constrained by local divergence. At the telomere side, we observed the pure *S. cerevisiae* telomeric repeats flanked by the Y' element. The Y' element origin is still

elusive and so far has only been found in species belonging to *Saccharomyces* complex closely related to *S. cerevisiae*[27]. Surprisingly, we observed that the Y' element is present in the *Z. parabailii* genome and likely derives from a *Saccharomyces* species. The strains containing the region B, show both types of Y' sequence, with a subset of them having the *S. cerevisiae*-type and others the *Z. parabailii*-type (Supp. Fig. 8a). Taken together, these results support a complex multi-step HGT scenario with the Y' element from a *Saccharomyces* species that initially invaded the *Z. parabailii* genome and inserted at multiple subtelomeres and was secondarily transferred in *S. cerevisiae* together with the flanking region B. It is possible that the primary transfer of Y' in *Z. parabailii* played some role in the mobilization, insertion and stabilization of the HGT associated chromosome end.

We previously proposed region G as a new large HGT with the peculiarity of being present in a single wild strain isolated from Ecuador from an unknown source[11]. Indeed, the *de novo* assemblies confirm the presence of region G at the chromosome XIII-R subtelomere of the haplotype 2 of the CLQCA_20-060 (ALI) strain (Extended Data Fig. 8, Supp. Fig. 8b). Similarity search of the protein coding sequence supports an undetermined *Lachancea* species being the region G donor.

Finally, while all the large HGT regions are present at telomeres, complete assemblies illustrate how these regions can evolve and re-localise in the core chromosome regions. This dynamic is perfectly exemplified by the region F (60 kb), which we detected both in long-subtelomeric and short-internal configurations in two unrelated strains (Extended Data Fig. 8, Supp. Fig. 8c). The most parsimony scenario is that the long-subtelomeric is ancestral and the short-internal is the evolved state that shortened during internalization.

Overall, although the molecular mechanisms initiating the HGT remain elusive (e.g. uptake of naked DNA or transient hybrids/cytoduction), the complete assemblies have revealed a role for both sequence homology at the insertion site and the requirement for telomere-repeats type at the extremity that likely constrain the landscape of yeast HGTs.

Mutation in tDNA anticodons

One mutation resulted in the formation of a new tRNA species in a single strain, CBK isolated from an insect in Germany. This tRNA arose from a G to T point mutation

which transformed a tyrosine tDNA anticodon from GTA (tY(GUA), located on chromosome X) into a TTA nonsense suppressor (*SUP7*(UUA)). This *SUP7* gene has been previously detected in a clinical isolate, YJM421, where it contributed to a pair of Dobzhansky–Müller incompatibility by allowing it to read through the specific premature stop codon in *COX15*[28]. Surprisingly, this premature stop codon is not present in the mitochondrial genome of CBK. The second family underwent an anticodon mutation in CEQ_1a, an African Palm wine isolate, where it resulted in a synonymous change from a tL(CAA) into tL(TAA) on chromosome 1 (Supp. Table 14).

Furthermore, a surprising and unique case of genetic code change was found on both haplotypes of the MC9 (AIS) strain. This change resulted from a C to A mutation at position 35 in the original tR(ACG)K gene located on chromosome XI in the Sc part of the genome (between YKR026C and YKR027W), generated a novel $tRNA^{Arg}$ with an AAG anticodon that would translate the CUU-Leu codons, and possibly the CUC-Leu codons as Arginine instead of Leucine. There are 5 other copies of the original tR(ACG) gene that most likely ensure the correct decoding of the CGT-Arg codons. The CUU-Leu codons are probably also normally translated as Leucine by the 3 original copies of the tL(UAG) genes but a certain level of ambiguous translation is expected from the presence of this novel tR(AAG) gene.

The *Tsu4* transposable element is active in *S. cerevisiae*

We also looked for the *Tsu4* element, originating from a lineage related to the *S. uvarum* or *S. eubayanus* species[29], because a single and nearly complete copy of *Tsu4* has been identified once in a strain (245) isolated from a rum distillery in the West Indies[29]. The authors proposed that this element would have been firstly horizontally transferred from *S. uvarum* into *S. paradoxus,* as revealed by the high similarity between *Tsu4* elements in these two species and a patchy distribution in *S. paradoxus,* and then secondarily transferred from *S. paradoxus* into *S. cerevisiae*[29]. We found the *Tsu4* element in a strain, CEY647 (CQS_1a), isolated from a bat in French Guiana. Surprisingly, this strain has 9 complete copies scattered on 7 different chromosomes, demonstrating that this element is active in *S. cerevisiae.*

Supplementary Methods

This section details the pipelines used for genome assembly, haplotype phasing, genome annotation and SV detection.

Genome assemblies

Supp. Fig. 9 illustrates all steps carried out for nuclear and mitochondrial genome assemblies as well as haplotype phasing.

Nuclear chromosomes

Raw fastq files were treated with porechop (version: 0.2.4; github.com/rrwick/Porechop) to remove both adapters and barcodes , downsampled with Filtlong (v0.2.0; github.com/rrwick/Filtlong) for a maximum of 40X per strain then assembled by both Canu (version: 2.0)[30] and SMARTdenovo (version: 5cc1356; github.com/ruanjue/smartdenovo). This generated two assemblies per strain that were treated separately. The contigs were polished using the same fastq data used for assembly. First both the Canu and SMARTdenovo assemblies are polished by Racon (version: 1.4.3) for one and three rounds respectively[31]. This is followed by two rounds of Medaka (version: 1.2.2; github.com/nanoporetech/medaka). The Nanopore polished contigs were then polished using Illumina data with Pilon (version: 1.22) for three rounds[32]. The hybrid-polished contigs were scaffolded against the reference genome using Ragout (version: 2.2)[33], and then manually curated to correct for un-scaffolded or badly scaffolded contigs, to generate a haploid assembly. Potential haplotigs and mitochondrial contigs were then removed based on a reference-based renaming of contigs/scaffolds. Assemblies were descaffolded and manually inspected for negative gaps using Gap5's 'Find internal joins' option (version: 1.2.14)[34]. Contig overlaps were considered negative gaps if the same two contigs were previously scaffolded in this gap and if the overlap was greater than 10kb. In these cases, the negative gaps were closed by generating an overlap consensus. Those assemblies that had negative gaps removed were then re-polished by Pilon once to correct for any errors introduced during the consensus generation step. Finally, assemblies were then re-scaffolded with Ragout (version: 2.2) followed by manual curation, generating the final assemblies.

For all haploid/homozygous and haplotype-collapsed assemblies, the two alternate

versions of each assembly (Canu and SMARTdenovo) allowed us to evaluate possible misassemblies or assembly artifacts by aligning them against the S288C reference genome as well as against one another using MUMmer's nucmer/mummerplot tools (version: 4.0.0beta2)[35] and manually inspecting for structural differences. In the case where a structural discrepancy was found between the Canu and SMARTdenovo versions, the best assembly corresponded to the one with the reference-like structure. Indeed, both assemblies are *de novo* structures and therefore an assembly sharing a reference-like chromosome structure is more likely to be real than the other uniquely rearranged assembly. Discrepancies in both assemblies were present only in heterozygous strains, with the exception of three homozygous/haploid strains (BAM, BGP and CRB). Due to this, the 'best' assembly for BAM and BGP contains a combination of contigs from Canu and SMARTdenovo (BGP contains all but chromosome V from the SMARTdenovo assembly and BAM contains primarily Canu except for chrXII, chrIII and chrVII_IV from SMARTdenovo). The CRB discrepancy was unresolved and therefore the choice of the best assembly relied on the assembly statistics below. In the absence of assembly structure discrepancies, assemblies were compared using basic genome statistics and those generated by MUMmer's dnadiff tool (version: 4.0.0beta2)[35]. We used the following values for comparison; number of scaffolds, number of contigs, percentage of reference covered, percentage of reference identity and genome size, in order of decreasing weight. The aim was to select a genome with an ideal number of 16 contigs/scaffolds and maximize the remaining values. Contig count was reduced by one if a scaffold was present within the rDNA array on chrXII. Selecting the 'best' phased assembly for polyploid strains relied purely on stats due to regular structural discrepancies between assemblies. Diploid heterozygous strains also contained structural discrepancies, however, the 'best' assembly relied upon phasing statistics (see Haplotype phasing).

Mitochondrial chromosomes

The mitochondrial *de novo* genome assemblies were constructed from the Illumina paired-end reads with a pipeline derived from ref[36]. Short reads were down-sampled to sets of 500,000, 600,000, 700,000 and 800,000 paired-end reads with seqtk (version: version: 1.3-r106; github.com/lh3/seqtk). Each dataset was *de novo* assembled with A5-miseq (version: 20160825)[37] and mitochondrial contigs were identified through similarity searches to the *S. cerevisiae* mitochondrial reference

sequence (accession number KP263414.1). For each strain, a representative assembly was selected based on the number of contigs and their length. The one-contig assemblies were subjected to Circlator (version: 1.5.5) for circularization[38]. For the non-circularisable ones, long reads were screened and used for manual circularisation if possible. A custom python script was used to set the starting position of the sequence to the ATP6 gene initiation codon.

Haplotype phasing

In order to test the phasing pipeline[39] a test dataset was generated from a strain, SO002, generated in the lab by crossing two stable haploids, YLF161 and YLF132[40], from West African (DVBPG6044) and North American (YPS128) backgrounds respectively. After generating the diploid, it was then sequenced by nanopore and illumina as with other strains within this study. This dataset was created to remove biases from generating diploids artificially through *in silico* read subset merging and as both parental strains have reference quality assemblies for comparison[8]. The results of the pipeline, the analysis, the results and raw data can be found at (https://github.com/SAMtoBAM/PhasedDiploidGenomeAssemblyPipeline).

For the 21 diploid heterozygous strains, the phasing pipeline was performed three times per strain, with each run only changing the genome backbone used for read alignment. The three backbone genomes used were the S288C reference genome and both Canu and SMARTdenovo collapsed *de novo* assemblies. Illumina reads were aligned to the genome assembly using BWA-MEM (version: 0.7.17)[41], converted to bam and sorted with SAMtools sort (version: 1.11)[42]. Next, variants were called using GATK MarkDuplicatesSpark and HaplotypeCaller (version: 4.1.8.1)[43] and filtered for a minimum mean DP of 30 with VCFtools (version: 0.1.5)[44]. ONT reads were aligned to each assembly using Minimap2 (version: 2.13)[45], converted to bam and sorted with SAMtools sort (version: 1.11). The resulting short-read based VCF and LR based BAM were given as input to Whatshap's phase tool (version: 1.0)[46] to phase the variants and produce a phased VCF. The phased VCF and LR based BAM were provided as input to Whatshap's haplotag tool to phase the reads and produce a phased BAM. Reads are then split by the Haplotype tag (HP1/HP2) and combined with unphased reads to generate one set of reads for each haplotype.

Phasing statistics were evaluated in order to determine the best assembly for phasing and in turn the best *de novo* assembly for each strain. In order to evaluate phasing

contiguity new phasing statistics were created, the V90 and the nV90. The V90, inspired by the L90 and N90, is the minimum number of phased blocks to cover 90% of all phased variants. The nV90 is the normalized V90, dividing the V90 by the total number of contigs in which the V90 blocks are present. Therefore, the ideal scenario is that each block corresponds to a unique contig giving an nV90 of 1. The highest nV90 was used to select the 'best' assembly. In 3 cases where the nV90 were equal in both Sdn and Canu assemblies, the assembly with the greatest total sum of phased base pairs was chosen. First, comparing the use of the *de novo* assemblies versus the reference, identified that *de novo* assemblies consistently performed better (Supp. Fig. 10). Secondly, out of the two *de novo* assemblies, the phasing results determined the 'best' assembly and the phased reads were used downstream for haplotype assembly. 6/21 genomes chosen based on phasing stats disagreed with the assembly choice based on stats alone.

For polyploid strains, we used the nPhase tool (version: 1.1.3) with default parameters to phase each polyploid using both long and short reads[47]. Once we obtained raw results using the nPhase pipeline command, we ran the nPhase cleaning command using default parameters to improve contiguity and eliminate short, uninformative haplotigs.

Genome annotation

Supp. Fig. 11 illustrates all steps carried out for nuclear and mitochondrial genome annotation.

All nuclear and mitochondrial assemblies were annotated with the LRSDAY pipeline (version: 1.6.0)[48]. Based on sequence homology comparison, LRSDAY automatically identifies protein-coding genes, centromeres, transposable elements, telomere associated X'- and Y-elements as well as important mitochondrial RNAs. Three types of transposable elements were annotated: complete and truncated Ty, as well as solo LTR, each of them being classified in Ty1, Ty2 (or Ty1/Ty2 for solo LTR), Ty3, Ty4 and Ty5 families. For each input assembly, the annotation of different genomic features were combined into a single GFF3 file, which were further examined, resorted, and verified by GFF3toolkit (version: 2.1.0). Those annotated protein coding genes with disrupted ORFs were labeled as pseudogenes during this process.

SV detection

Supp. Fig. 12 illustrates all steps carried out for SV detection.

The script[49] for generating the non-redundant SV dataset is available on github (https://github.com/SAMtoBAM/MUMandCo/tree/master/nonredundant_population_datasets). MUM&Co (version: 3.8)[50] called SVs separately on all genomes generated, including both Canu and SMARTdenovo, both phased and collapsed, 4 complex aneuploid chromosome assemblies and 23 public assemblies, totalling 3,229 assemblies. For phased polyploid genomes, each individual phased block assembly was run separately and the -ml/--minlen was increased to 100. All raw calls were aggregated and then both insertion events involving scaffold fragments (N's) and calls within the region surrounding the rDNA cluster on chrXII in the reference (position 450-490 kb) were removed. This gave a total of 95,893 raw, unvalidated calls. For any call to be validated, and aggregated into a non-redundant call, it had to match the following criteria with another call of the same type in another assembly: for Deletions, Duplications and Contractions events: matching start and end reference position +- 300 bp; for Insertion events: matching start and end reference position +- 300 bp and Size of inserted fragment (+-10%); for Inversion events: matching start and end reference position +- 6.5 kb; for translocation events: matching start and/or end reference position +- 6.5 kb and matching chr involved at border and matching position within chr involved at border +- 6.5 kb.

If any calls did not match another call using these rules, it was removed. Therefore, validation requires a call to be found within at least two genome assemblies, whether within or between strains. An exception is made for both duplications and contractions which can be validated within a single genome if the region contains more or less than one additional copy, i.e. triplicated, and therefore contains multiple duplication calls for a single region. However, there was only one occurrence of a duplication being validated within only a single assembly. After the aggregation of calls into a single non-redundant event, an average value for the positions and size was taken. Furthermore, the orientation for translocation events (following border events from VCF formatting, e.g. [chrI:1[) was taken by consensus. In the case that there was an equal number of both orientations, either contigs were reverse complemented followed by re-calling SVs (commonly found in the case of polyploid phased assemblies that were not reference orientated prior to SV calling) or calls were split in the case of likely over-aggregation. Additionally, due to each phased block for polyploid strains having been

analyzed for SVs individually, the discontinuity of each ‘assembly’ added large false positive deletions. Due to this, all large deletions up to a size of 20 kb were removed if not manually verified by coverage analysis, as performed for aneuploidy detection (refer to Aneuploidy detection). Using these rules, 91,645 (95.6%) were considered validated, which when split by SV type ranged from 78-99% for translocations and contractions, respectively (Supp. Fig. 13). Additionally, an average of 95.7% of all variants within each strain were validated. Notably however, genomes that were not assembled within the frame of this project had a lower average within-strain validation due to only containing a single assembly per strain. Furthermore, many from Bendixsen et al. (2021)[2] have genetically diverse East Asian origins and therefore reducing the likelihood of sharing an SV with another strain within this cohort (Supp. Fig. 14). The total non-redundant dataset contains 4,809 SVs.

For diploid genomes, validated SVs were considered homozygous if found in at least one genome from both HP1 and HP2 for the same strain. Alternatively, SVs were considered heterozygous if only a single haplotype (HP1 or HP2), from the same strain, were found to contain the SV. Because SV validation requires a call to be found within at least two genome assemblies, the additional calls validated due to phasing correspond more to heterozygous variants due to the disentangling of their otherwise collapsed status. In this respect, there is an assumption that the phased genome contains two complete haplotypes due to the fact that unphased reads were also added to phased diploid genome assemblies. In contrast, polyploid phased genomes contain only phased reads and therefore the number of haplotypes can vary for any reference position. Therefore, in order to label calls homozygous or heterozygous, first the number of haplotypes must be estimated. Polyploid phased genomes were first aligned to the reference using Minimap2 (version: 2.17; options: -ax asm5 --secondary=no) and coverage per reference base pair calculated. The number of haplotypes in regions of deletions and contractions was determined by the median coverage 20-kb up and downstream of the event. For all other events, the number of haplotypes was calculated using the median coverage of both 20-kb up and downstream and within the event itself. All SVs that were present within fewer phased assembly blocks than the number of predicted haplotypes were considered heterozygous. Lastly, in regions where the number of predicted haplotypes was greater than the ploidy +2 (e.g. 5 in a triploid), events were considered homozygous.

As the distinction between homozygous and heterozygous genomes was solely based on SNVs, we checked that the lower number of SVs in SNV-based homozygous genomes was not due to a lack of detection of heterozygous SVs because SNV-based homozygous genomes were not phased. We ran Sniffles (version: 2.0.2)[51], a read-based SV detection tool, which is therefore not prone to missing heterozygous variants. We also found more SVs relative to SNVs in heterozygous strains, and found no evidence that any significant number of heterozygous SVs were missed in our homozygous strains (Supp. Fig. 15a). Furthermore, the same association was found using paftools (version: 2.17)[45], another assembly-based tool using minimap2 mapping as opposed to nucmer (Supp. Fig. 15b). These analyses show that the higher number of SVs found in heterozygotes as compared to homozygotes is not due to a methodological bias.

Insertions, deletions, duplications and inversions can be further broken down into whether they involve novel or repetitive regions of the genome. To calculate the material involved in each SV was searched using BLAST (version: 2.2.31) against the reference. In order for an insertion to be novel, the material should not already be present. However, for deletions, duplications and inversions, they by definition are already present and therefore are considered novel if only present once. Therefore, after the BLAST search, matches were filtered to have an e-value <0.001 and a base identity >90%. The sum of the remaining lengths was then calculated. This sum needed to be above 50% or 150% of the query length to be considered a repetitive region in insertions and deletion/duplications respectively.

One way to look at gene impact is to categorize the type of impact. Four categories have been created, ignorant of the resulting ORF(s) and its/their direction: (i) Contained = At least one entire gene is contained within the SV (excludes insertions and translocations), (ii) Disruption = A single gene is present at the border of an SV (excludes insertions), (iii) Within = the entirety of the rearrangement is confined within a single gene (excludes translocations) and (iv) Fusion = Both borders of an SV interact with different genes which would therefore bring them into frame in a way (excludes insertions). The categories are not mutually exclusive as a single event can both contain entire genes and disrupt/fuse others at the border. However, an event cannot disrupt and fuse and/or be within. Additionally, the gene repertoire can be filtered to only contain essential genes and the analysis repeated. However, fusion

can happen between essential and non-essential genes. Therefore, a 5th category in a way was added: (v) Fusion-between-essential-and-nonessential = Both borders of an SV interact with different genes which would therefore bring them into frame in a way (excludes insertions). One of those genes is an essential gene.

Supplementary Figures

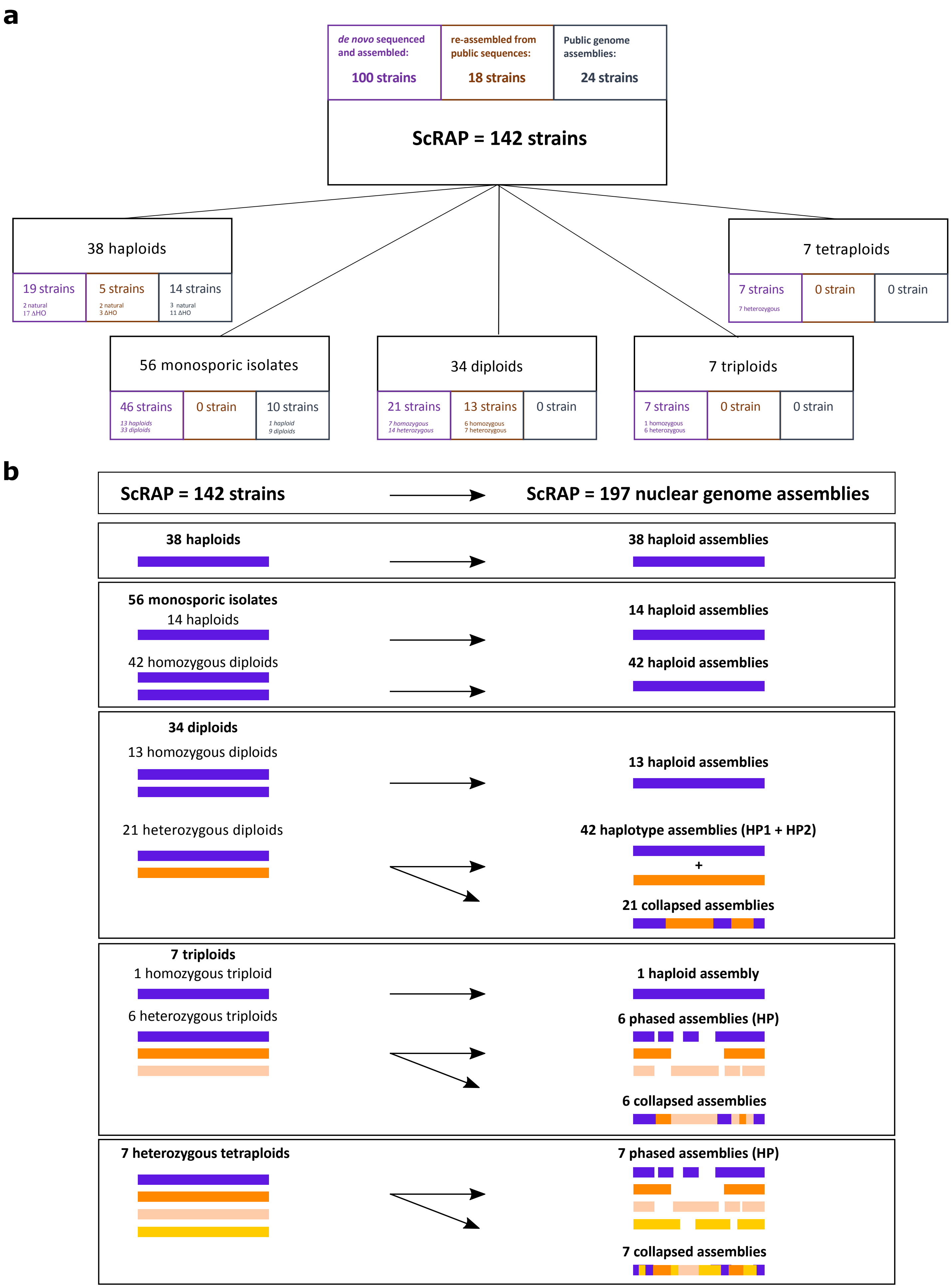

Supplementary Fig. 1: Description of the 142 strains belonging to the ScRAP. **a.** The strains come from 3 datasets, (i) 100 de novo sequenced and assembled genomes, (ii) 18 re-assembled genomes using previously available raw Nanopore read data and (iii) 24 publically available complete genome assemblies, including the S288C reference genome. The number of strains with different ploidy and heterozygosity levels is indicated for each of the three datasets. b. b. correspondence between the strain ploidy and zygosity and the number and type of genome assemblies.

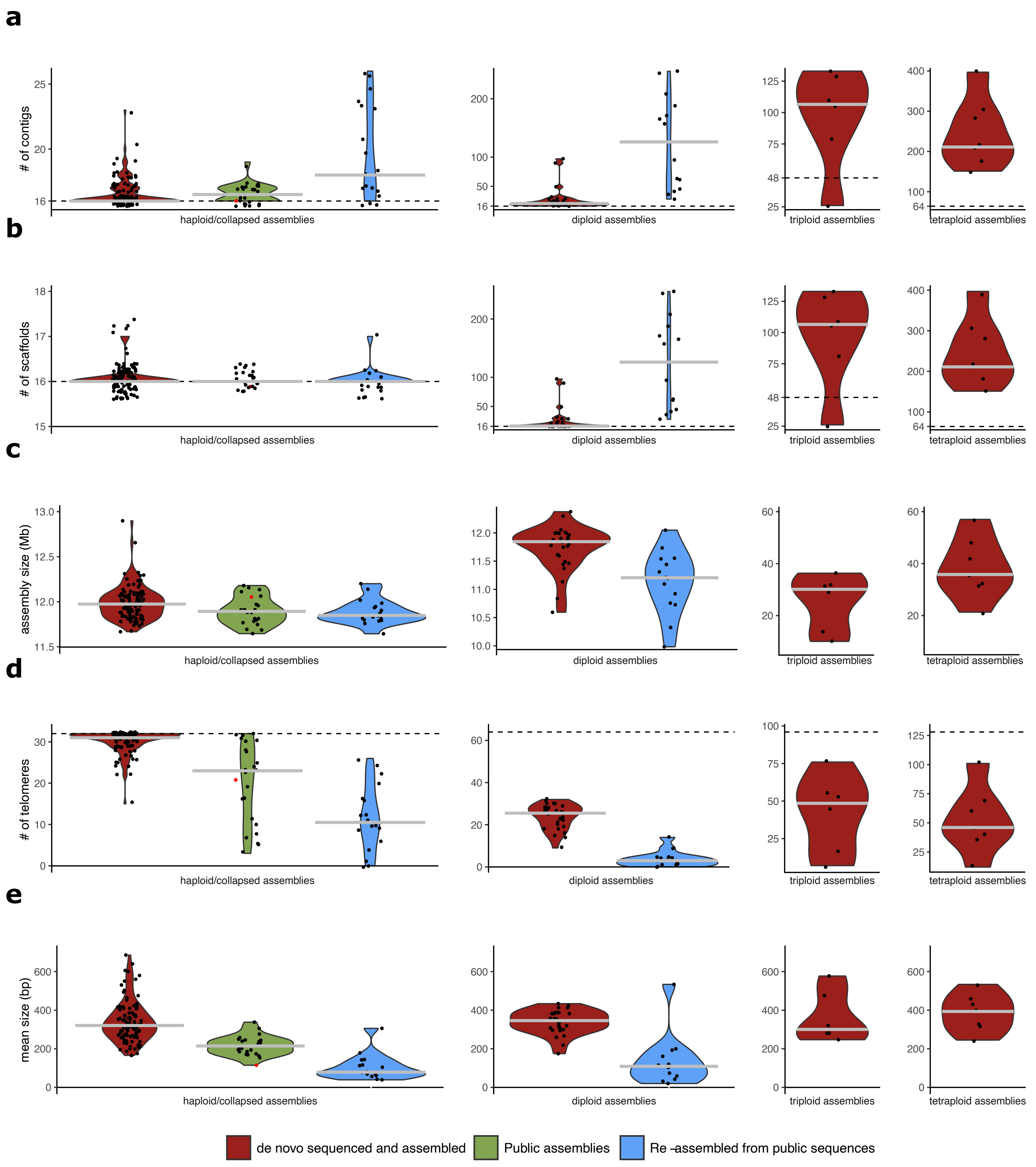

Supplementary Fig. 2: Distribution of various assembly metrics. Each black dot corresponds to the value for one strain, the red dot shows the position of the S288C reference genome in the distributions. Median values are indicated by the gray crossbars. The horizontal dotted lines show the target values for complete assembly. For Haploid/collapsed assemblies: n=100 de novo sequenced assemblies, n=18 re-assemblies and n= 24 public assemblies. For diploid assemblies: n=28 de novo sequenced haplotype assemblies and n=14 haplotype re-assemblies. For triploid and tetraploid assemblies: n=6 and n=7 de novo assemblies, respectively. **a.** Number of contigs per strain. **b.** Number of scaffolds per strain. **c.** Genome assembly size. **d.** Number of telomeres per strain. **e.** Mean telomere size per strain.

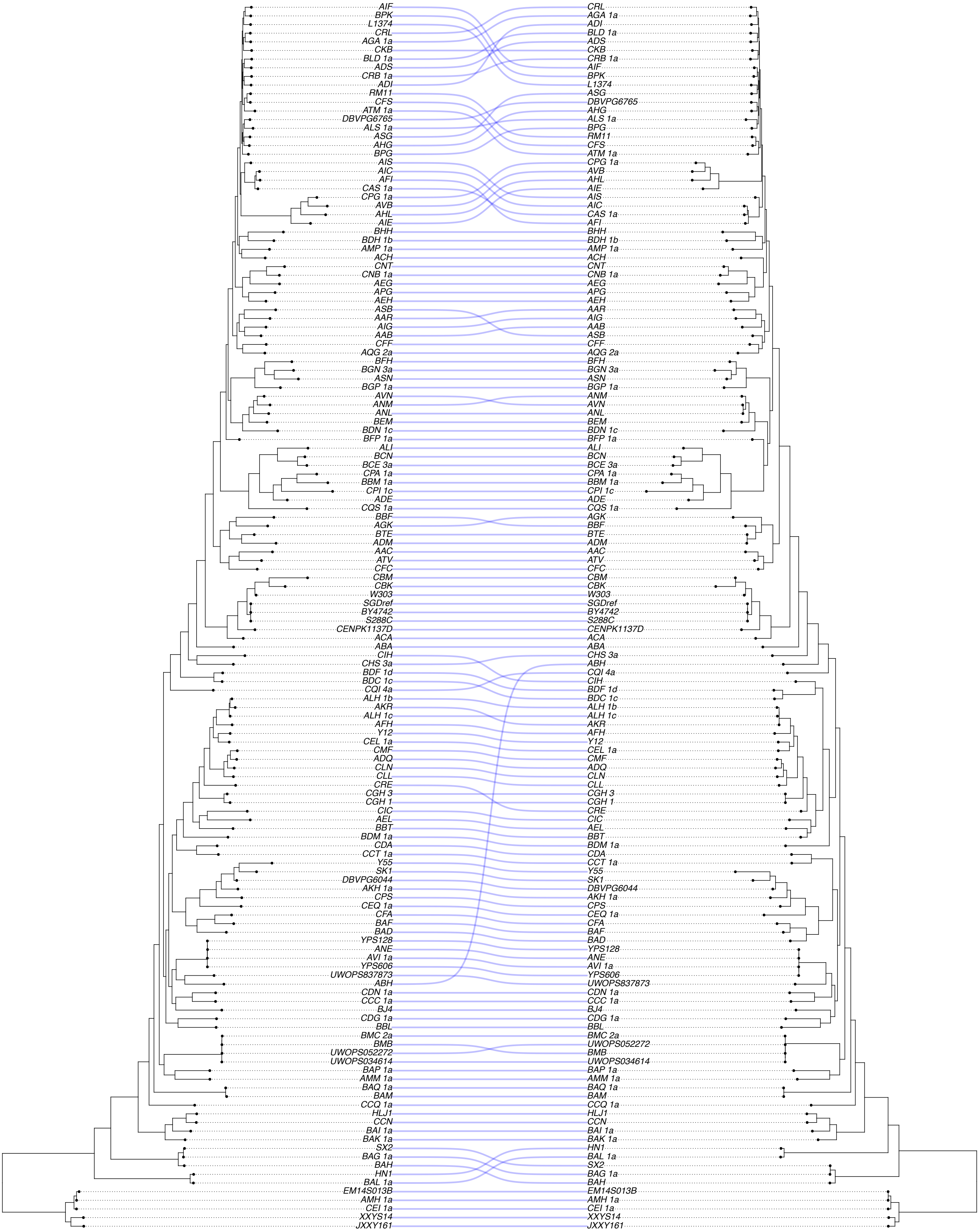

Supplementary Figure 3: Comparison between phylogentic trees. The ortholog-based tree is on the left and the SNP-based tree is on the right.

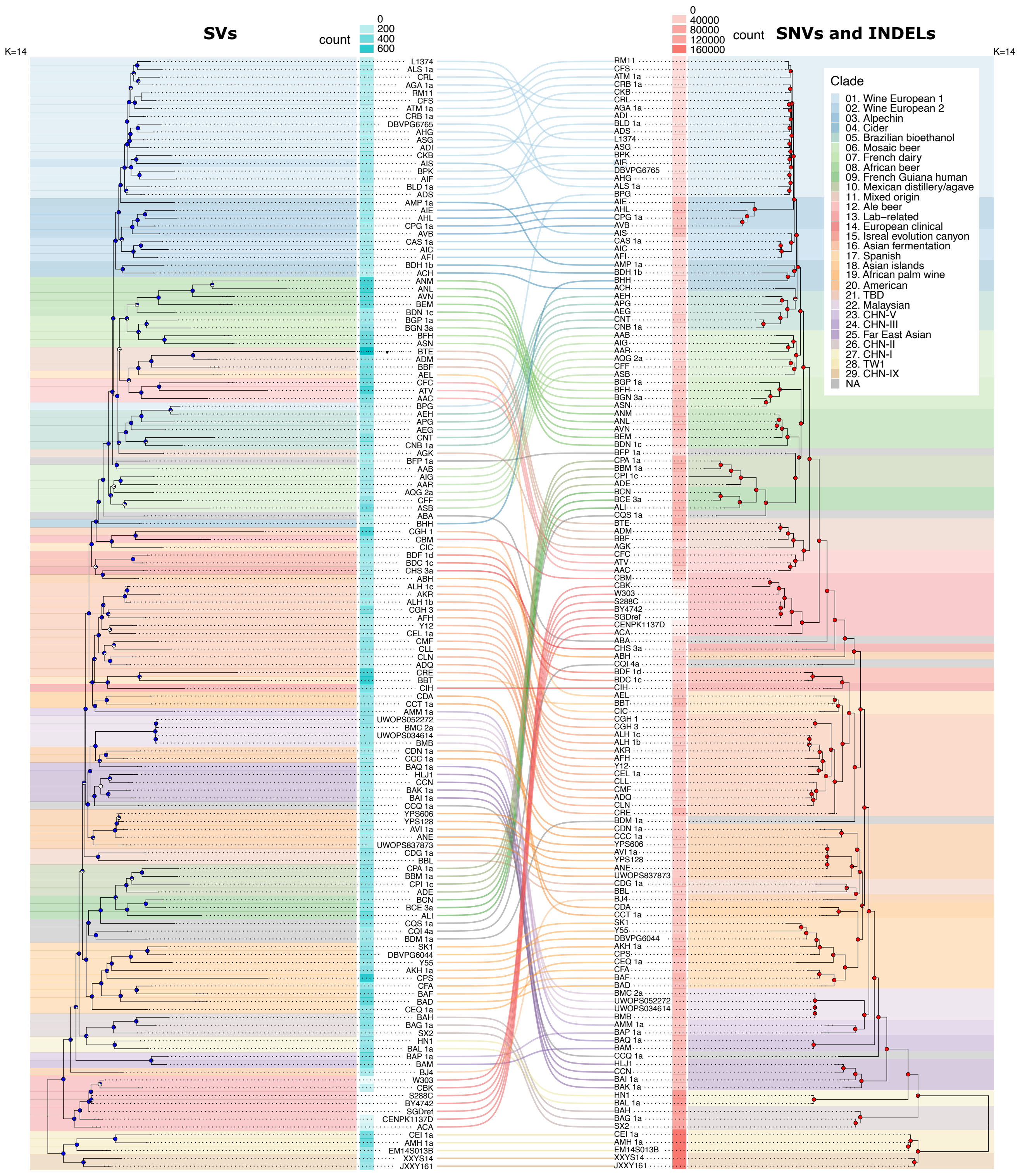

Supplementary Fig. 4: Phylogenetic tree comparison. Comparison between the SV-based (left) and SNV/INDEL-based (right) phylogenetic trees. The genetic ancestry of each strain was inferred by ADMIXTURE using SVs (k=16) and SNVs/INDELs (k=14) and the number of SVs and SNVs/INDELs per strain is presented as cyan and coral heatmaps, respectively.

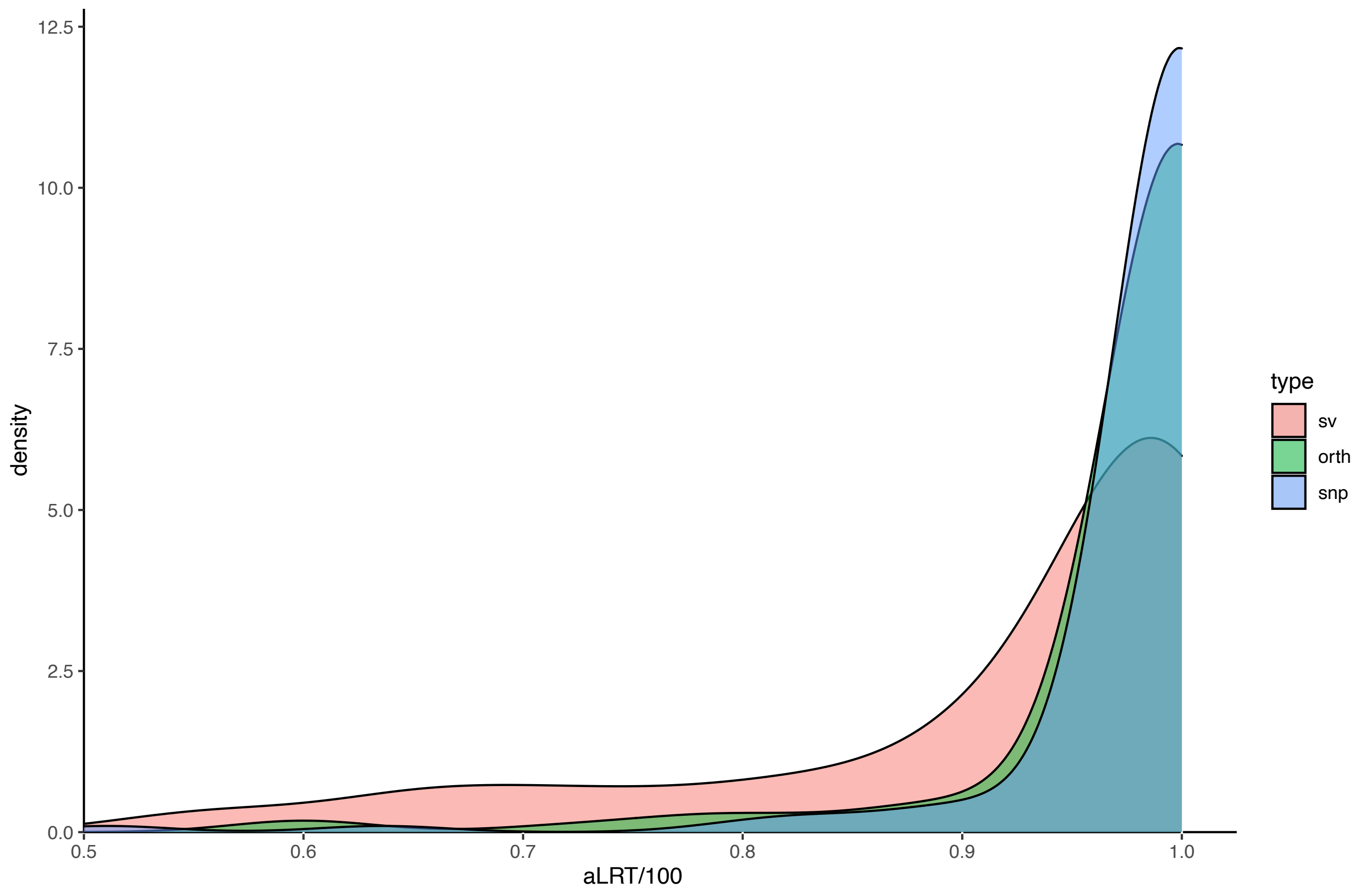

Supplementary Fig. 5: Distribution of node support values. Comparison of the distributions of the aLRT node support values between the orthologue, SNP and SV-based phylogenetic trees.

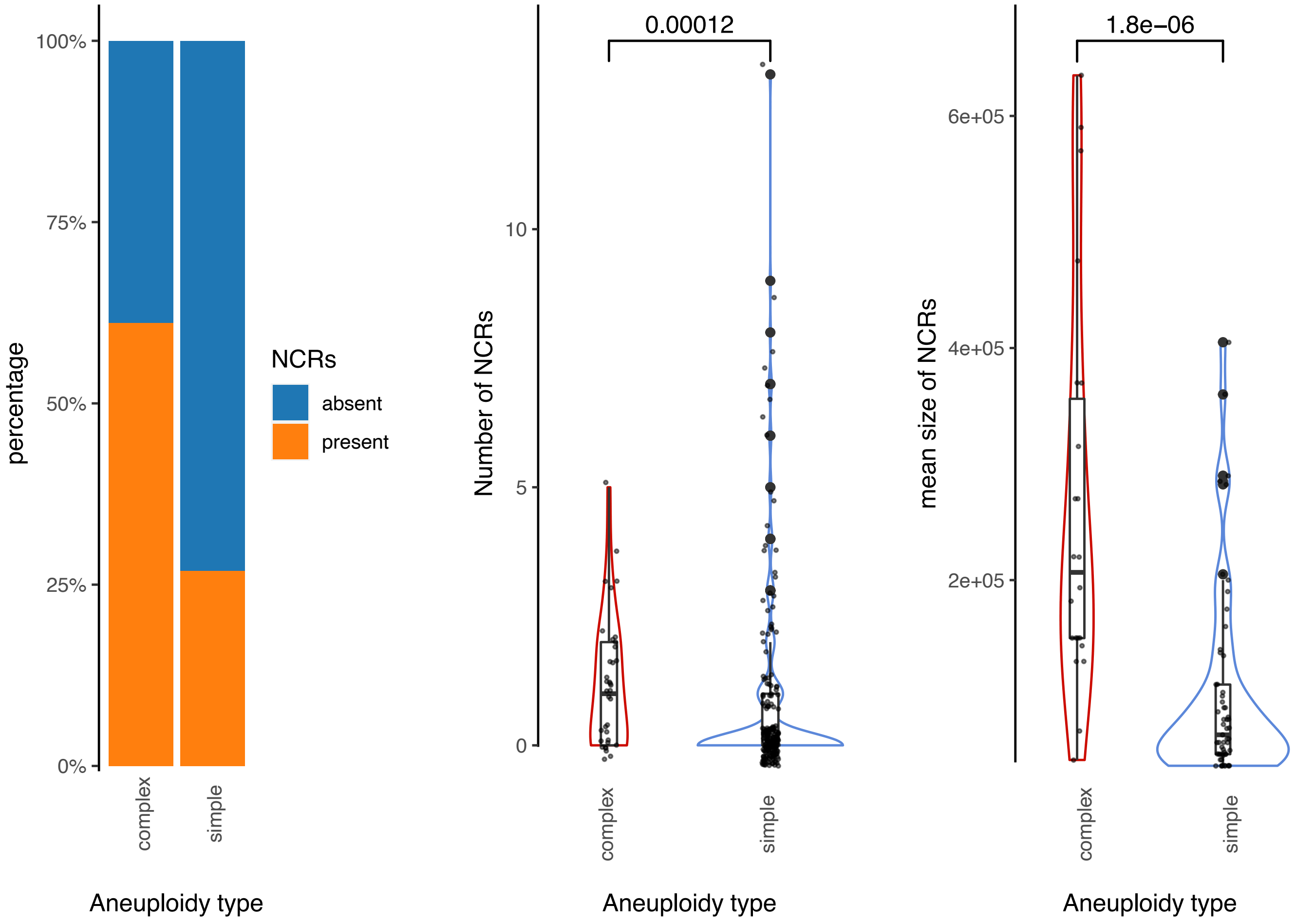

Supplementary Fig. 6: Contribution of Non-Centromere Related (NCRs) coverage deviations in strains containing simple and complex aneuploidies. Strains with complex aneuploidies are more likely to have an NCR (left), are more likely to have a higher number of them (middle) and are more likely to have on average a larger (mean size) NCRs (right). The horizontal lines in the boxplots (derived from n=36 complex and n=212 simple aneuploidies) correspond to the median, the lower and upper hinges correspond to the first and third quartiles and the whiskers extend up to 1.5 times the inter quartile range. Two-sided Wilcoxon mean comparison p-values are indicated.

a

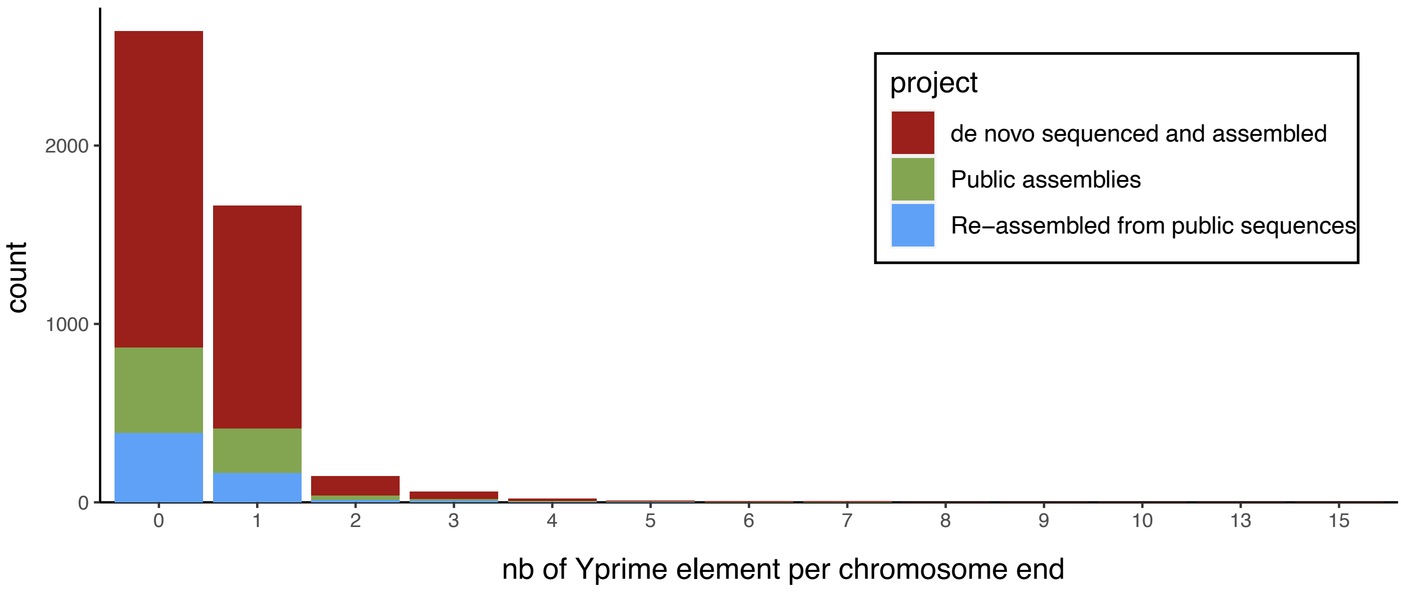

b

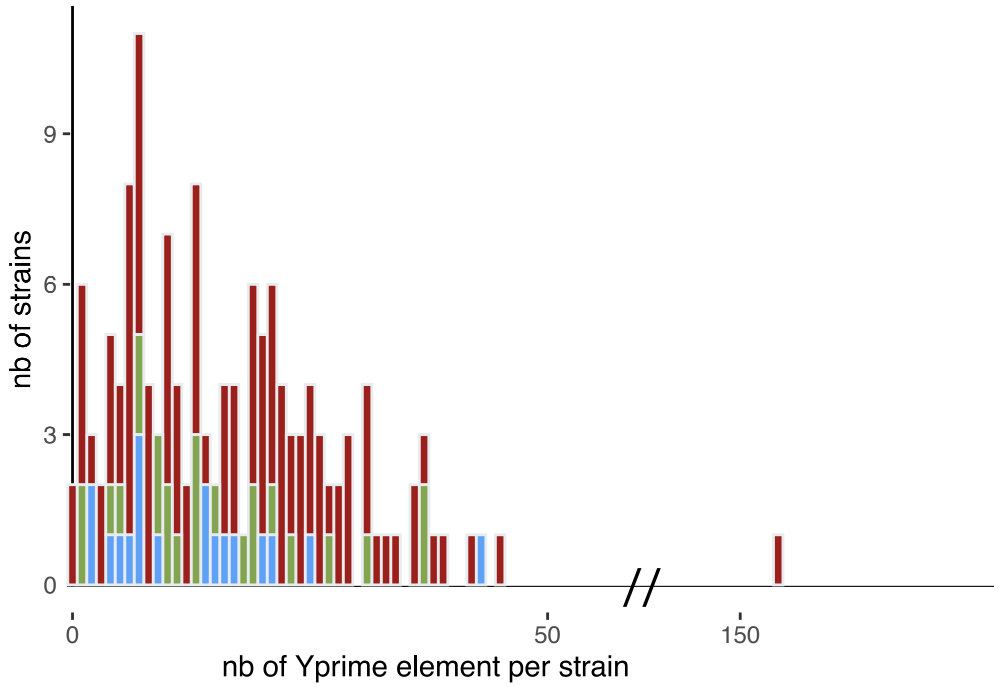

Supplementary Fig. 7: Distribution of the Y' elements in the ScRAP. **a.** Histogram of the number of Y' elements per chromosome end, split by dataset. **b.** Histogram of the number of Y' elements per strain, split by dataset.

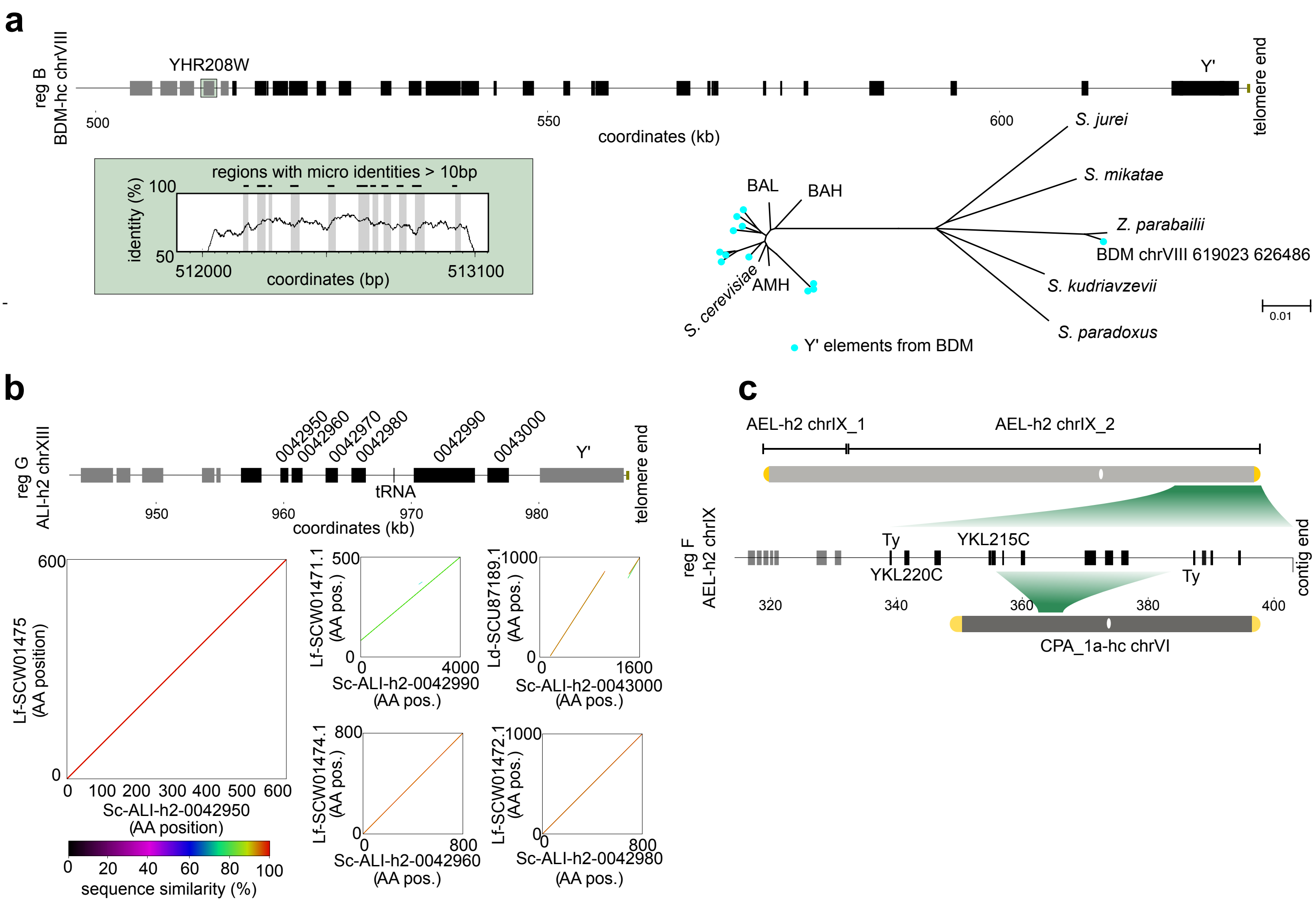

Supplementary Fig. 8: Characteristics of horizontally acquired regions. a. The HGT region B from Zygosaccharomyces parabailii was fully assembled in its native chromosomal position. Roughly 2 kb upstream the HGT (light grey boxes), we observed a 1 kb region (corresponding to YHR208W) with high local homology between S. cerevisiae and Z. parabailii (75% identical, overall), including several tracts between 10 and 20 bp of complete identity (green boxes). At the telomere side, this region shows a Y′ element, which we found also in the Z. parabailii assembly. The phylogenetic tree of the Y′ elements shows that the Y′ sequence in Z. parabailii is different from the one of the other Saccharomyces species and several copies of the Y′ BDM gave a close match, while other have an S. cerevisiae-type sequence. These data are consistent with a 3-way HGT exchange, with an initial transfer of Y′ (and perhaps associated sequences) from an undetermined species of the Saccharomyces genus to Z. parabailii followed by the transfer to S. cerevisiae. **b.** Although the ORFs from region G, identified only in the ALI isolate, had been already described in Peter et al 201832, long-read assemblies enabled to describe the region as a genuine HGT and to identify the donor species. Protein sequence alignments reveal several ORFs with high sequence identity, up to 100% amino acid (AA) identity and 100% overlap (i.e. ORF 0042950) with L. fermentati (Lf). Lower identity values are observed also in the alignments against L. dasiensis (Ld). **c.** Region F from an unknown source was retrieved in the assemblies of two strains, in the canonical subtelomeric position in AEL as well as reduced in core-chromosomal positions in CPA.

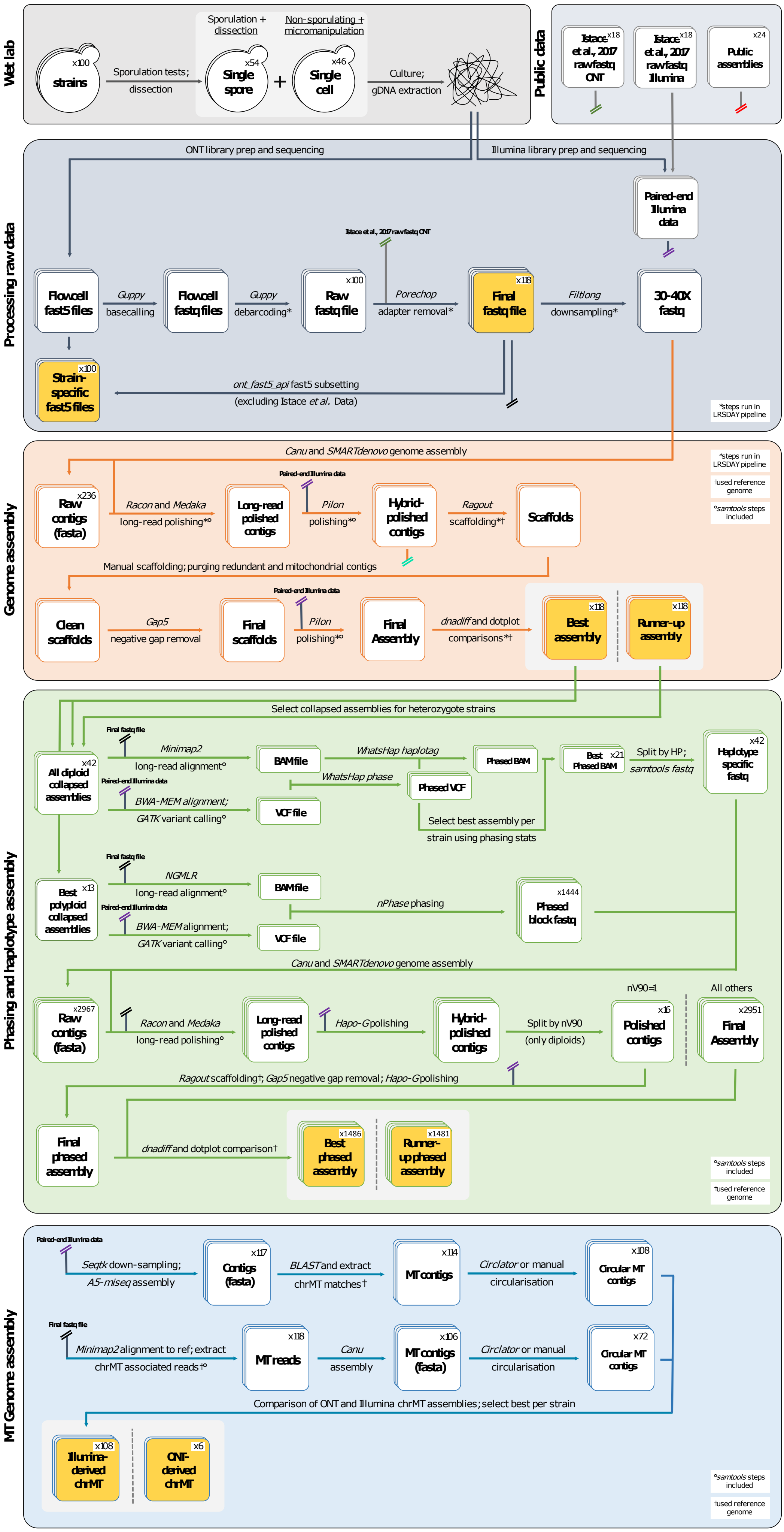

Supplementary Figure 9: Schematic representation of the assembly process. Yellow boxes indicate the the available files released in the frame of this work

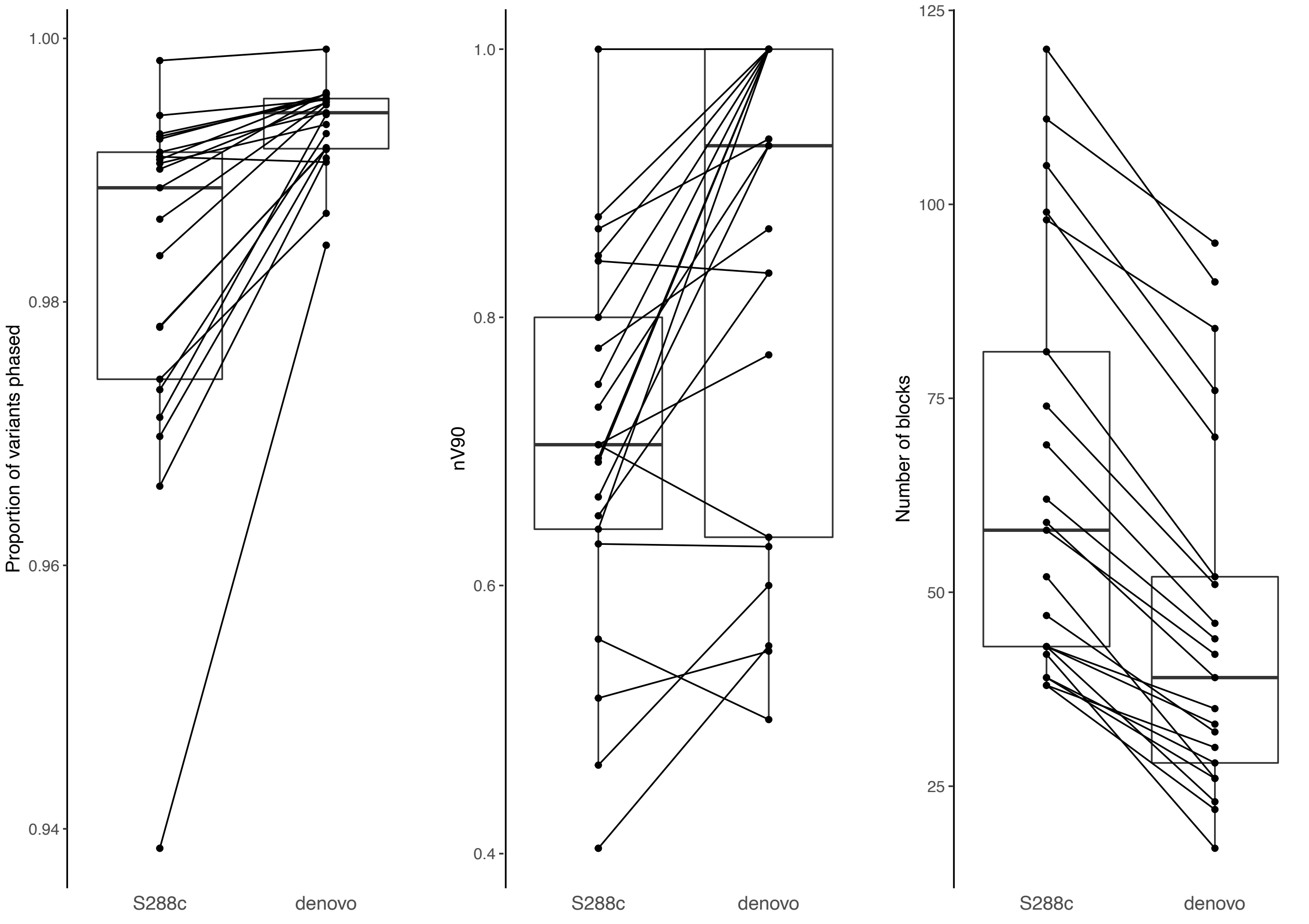

Supplementary Fig. 10: Comparison of diploid phasing results when using the S288C reference genome versus the de-novo assemblies as the reference. Three statistics are shown for comparison, the number of heterozygous variants that were phased, the nV90 and the number of phased blocks. All 3 metrics move in the direction expected for better phasing quality when using the de novo assemblies. The horizontal lines in the boxplots correspond to the median, the lower and upper hinges correspond to the first and third quartiles and the whiskers extend up to 1.5 times the inter quartile range (n=21 heterozygous diploid strains in each boxplot).

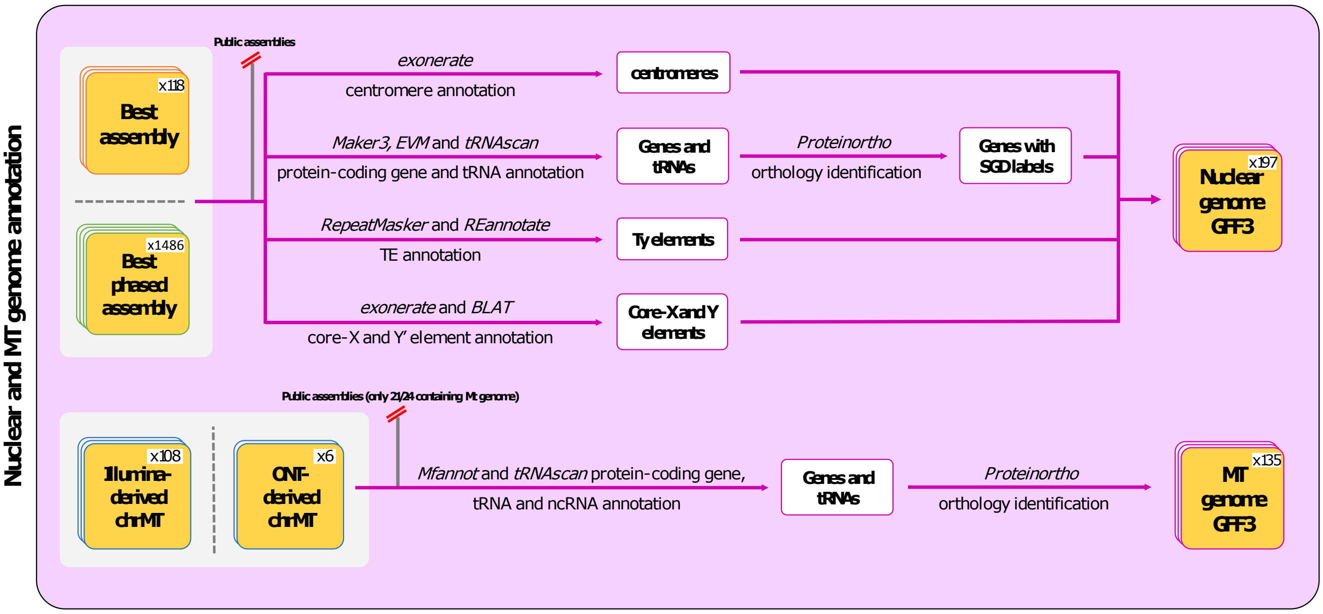

Supplementary Fig. 11: Schematic representation of the annotation process. All steps were run in the LRSDAY tool. The orthology detection step was run with proteinortho using the S288C reference genome annotation. Yellow boxes indicate the available files released in the frame of this work.

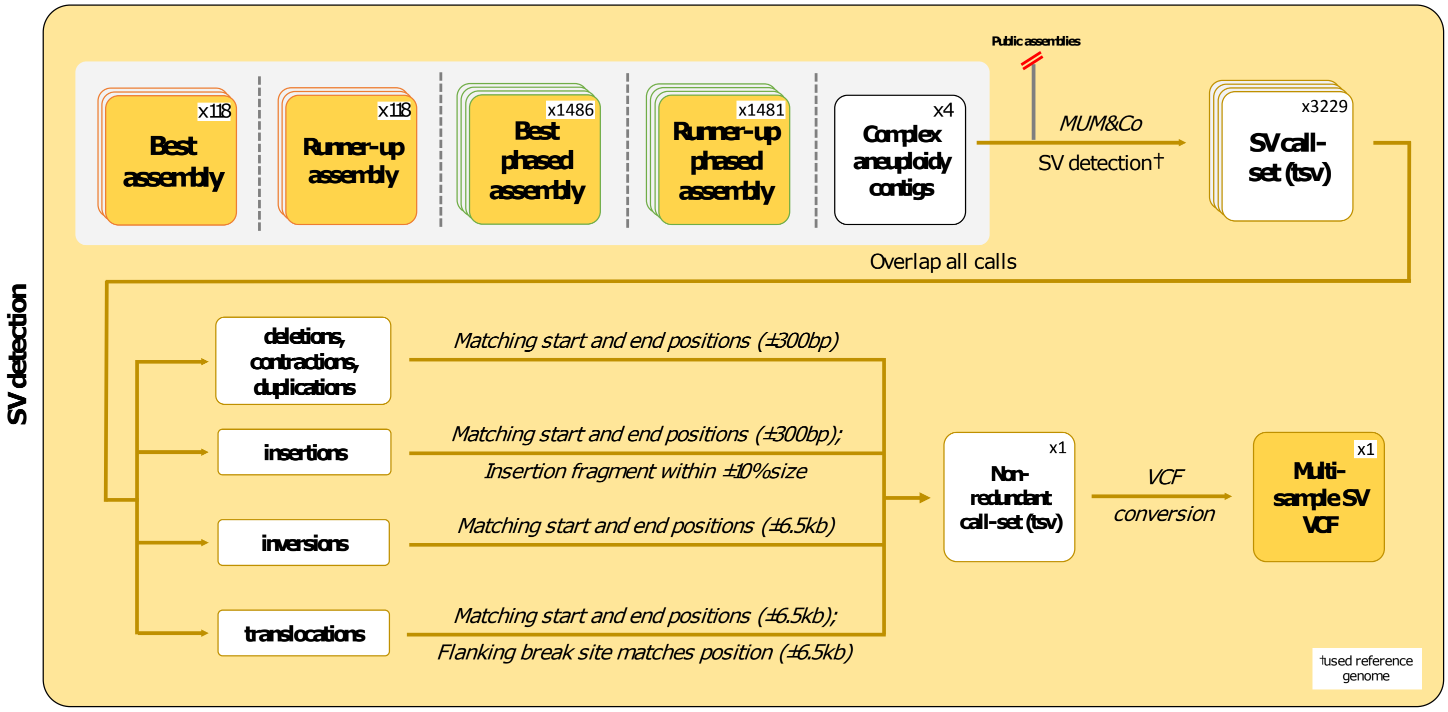

Supplementary Fig. 12: Schematic representation of the SV detection process. Yellow boxes indicate the available files released in the frame of this work.

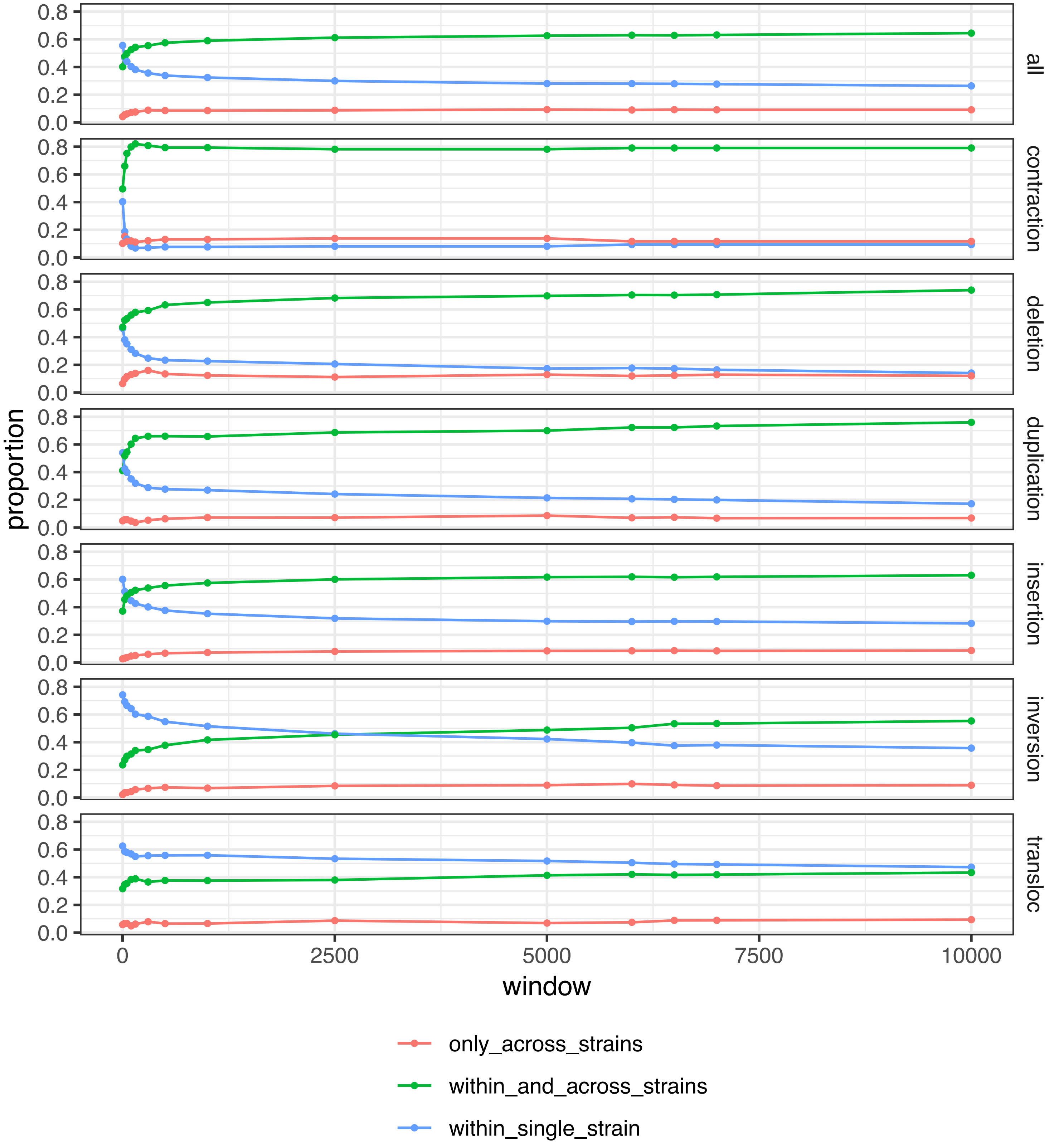

Supplementary Fig. 13: Validation of SV calls. Validation requires a call to be found within at least two genomes, whether within or between strains. The curves show the evolution of the proportion of validated calls as a function of the window size used to consider that two breakpoints were overlapping. The categories are as follows, the SV is only validated by genomes from the same strain (within single strain). The SV is validated by being found in multiple genomes for 1 or more strains, plus being found in multiple strains (within and across strains) and the SV is validated across strains without having multiple genomes for any of those strains (only across strains).

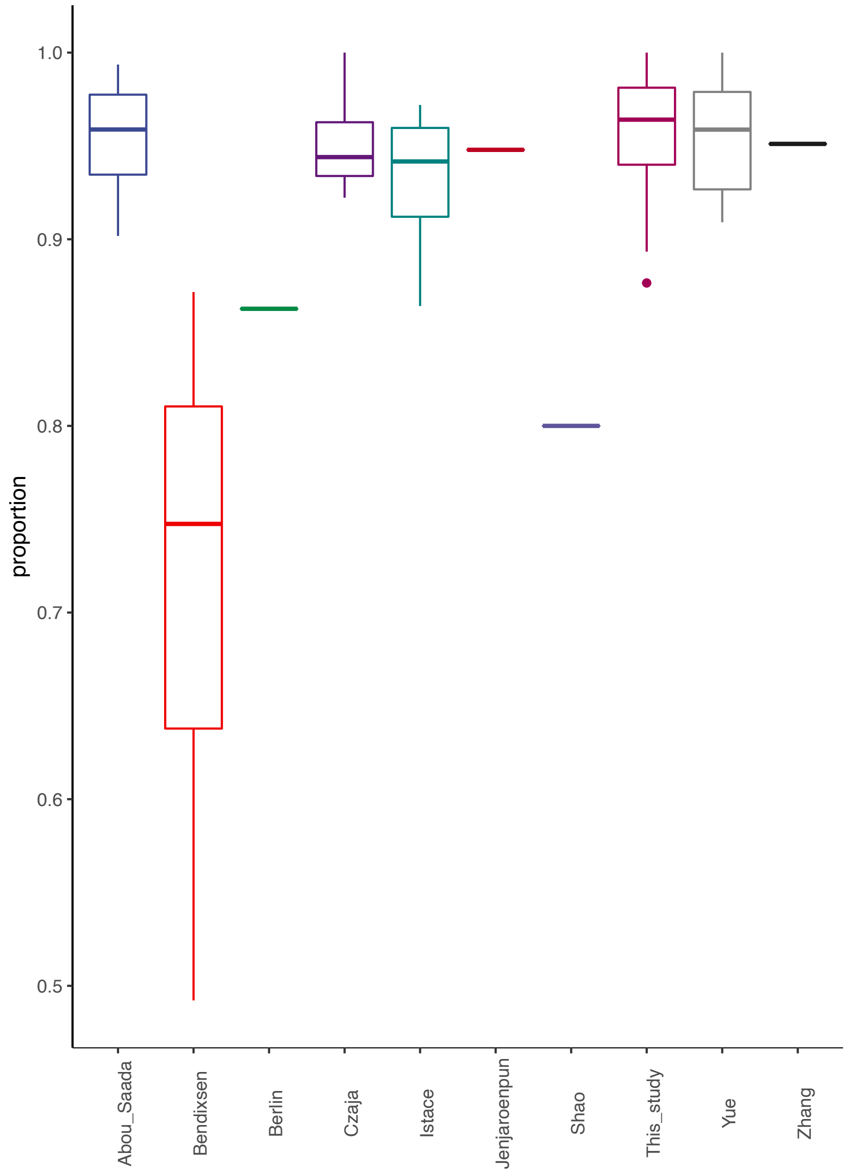

Supplementary Fig. 14: Proportion of validated SV split by publication. The names of the first authors of the following publications are indicated 1–4,6–9,46. The horizontal lines in the boxplots correspond to the median, the lower and upper hinges correspond to the first and third quartiles and the whiskers extend up to 1.5 times the inter quartile range. For each study, the number of assemblies in each boxplot is n=8 for Bendixsen, n=1 for Berlin, n=4 for Czaja, n=32 for gigascience, n=1 for Jenjaroenpun, n=4 for nPhase, n=137 for Phenovar, n=1 for Shao, n=7 for Yue and n=1 for Zhang.

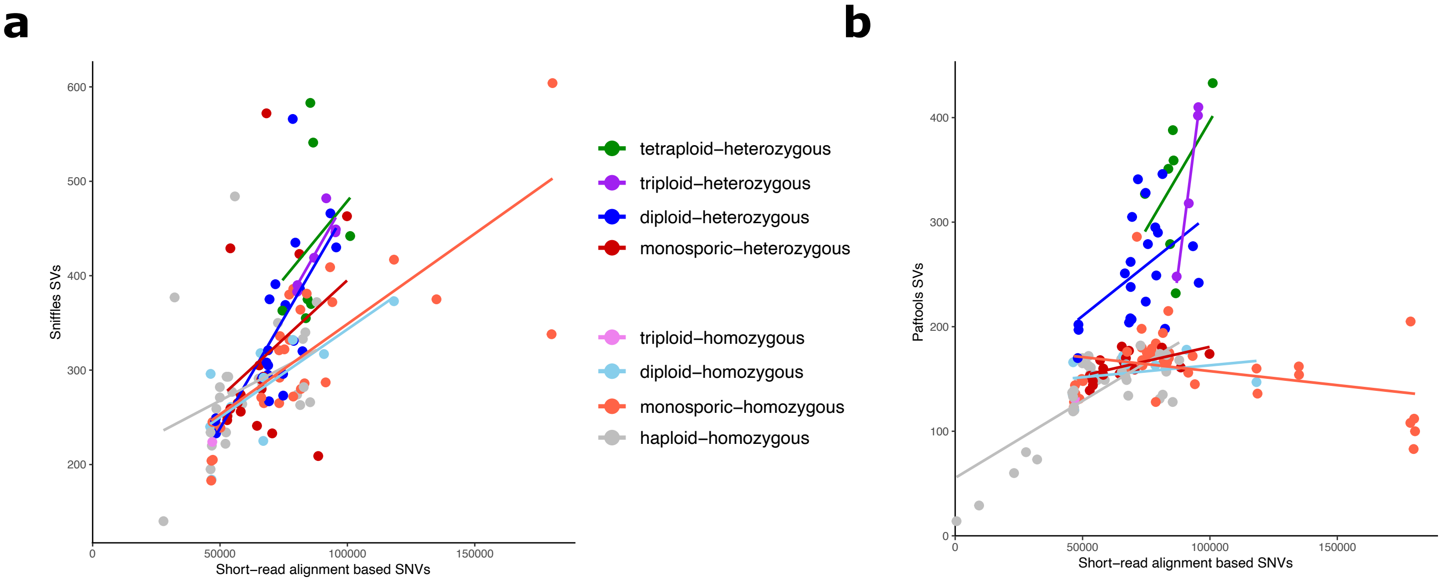

Supplementary Fig. 15: Correlation between SV and SNV accumulation. Total number of **a.** sniffles and **b.** paftools derived SVs as a function of SNVs per strain. The categories 'Heterozygous monosporic' and 'Homozygous monosporic' correspond to monosporic isolates derived from the sporulation of heterozygous and homozygous parental diploid strains, respectively.

Supplementary References

1. Istace, B. *et al.* de novo assembly and population genomic survey of natural yeast isolates with the Oxford Nanopore MinION sequencer. *GigaScience* **6**, (2017).
2. Bendixsen, D. P., Gettle, N., Gilchrist, C., Zhang, Z. & Stelkens, R. Genomic Evidence of an Ancient East Asian Divergence Event in Wild Saccharomyces cerevisiae. *Genome Biol. Evol.* **13**, (2021).
3. Berlin, K. *et al.* Assembling large genomes with single-molecule sequencing and locality-sensitive hashing. *Nat. Biotechnol.* **33**, 623–630 (2015).
4. Czaja, W., Bensasson, D., Ahn, H. W., Garfinkel, D. J. & Bergman, C. M. Evolution of Ty1 copy number control in yeast by horizontal transfer and recombination. *PLOS Genet.* **16**, e1008632 (2020).
5. Goffeau, A. *et al.* Life with 6000 genes. *Science* **274**, 546, 563–567 (1996).
6. Jenjaroenpun, P. *et al.* Complete genomic and transcriptional landscape analysis using third-generation sequencing: a case study of Saccharomyces cerevisiae CEN.PK113-7D. *Nucleic Acids Res.* **46**, e38 (2018).
7. Shao, Y. *et al.* Creating a functional single-chromosome yeast. *Nature* **560**, 331–335 (2018).
8. Yue, J.-X. *et al.* Contrasting evolutionary genome dynamics between domesticated and wild yeasts. *Nat. Genet.* **49**, 913–924 (2017).
9. Zhang, X. & Emerson, J. J. *Inferring the genetic architecture of expression variation from replicated high throughput allele-specific expression experiments*. 699074 https://www.biorxiv.org/content/10.1101/699074v2 (2019) doi:10.1101/699074.
10. Louis, E. J. & Borts, R. H. A complete set of marked telomeres in Saccharomyces cerevisiae for physical mapping and cloning. *Genetics* **139**, 125–136 (1995).
11. Peter, J. *et al.* Genome evolution across 1,011 Saccharomyces cerevisiae isolates. *Nature* **556**, 339 (2018).
12. D'Angiolo, M. *et al.* A yeast living ancestor reveals the origin of genomic introgressions.

Nature **587**, 420–425 (2020).

13. Duan, S.-F. *et al.* The origin and adaptive evolution of domesticated populations of yeast from Far East Asia. *Nat. Commun.* **9**, 2690 (2018).
14. Lee, T. J. *et al.* Extensive sampling of Saccharomyces cerevisiae in Taiwan reveals ecology and evolution of predomesticated lineages. *Genome Res.* gr.276286.121 (2022) doi:10.1101/gr.276286.121.
15. Wang, Q.-M., Liu, W.-Q., Liti, G., Wang, S.-A. & Bai, F.-Y. Surprisingly diverged populations of Saccharomyces cerevisiae in natural environments remote from human activity. *Mol. Ecol.* **21**, 5404–5417 (2012).
16. Alexander, D. H. & Lange, K. Enhancements to the ADMIXTURE algorithm for individual ancestry estimation. *BMC Bioinformatics* **12**, 246 (2011).
17. Strope, P. K. *et al.* The 100-genomes strains, an S. cerevisiae resource that illuminates its natural phenotypic and genotypic variation and emergence as an opportunistic pathogen. *Genome Res.* **25**, 762–774 (2015).
18. Selmecki, A., Forche, A. & Berman, J. Aneuploidy and isochromosome formation in drug-resistant Candida albicans. *Science* **313**, 367–370 (2006).
19. Shukla, A. *et al.* Chromosome arm aneuploidies shape tumour evolution and drug response. *Nat. Commun.* **11**, 449 (2020).
20. Taylor, A. M. *et al.* Genomic and Functional Approaches to Understanding Cancer Aneuploidy. *Cancer Cell* **33**, 676-689.e3 (2018).
21. Altemose, N. *et al.* Complete genomic and epigenetic maps of human centromeres. *Science* **376**, eabl4178 (2022).
22. Gilchrist, C. & Stelkens, R. Aneuploidy in yeast: Segregation error or adaptation mechanism? *Yeast* **36**, 525–539 (2019).
23. Beroukhim, R. *et al.* The landscape of somatic copy-number alteration across human cancers. *Nature* **463**, 899–905 (2010).
24. Mulla, W., Zhu, J. & Li, R. Yeast: A simple model system to study complex phenomena of aneuploidy. *FEMS Microbiol. Rev.* **38**, 201–212 (2014).

25. Bergström, A. *et al.* A High-Definition View of Functional Genetic Variation from Natural Yeast Genomes. *Mol. Biol. Evol.* **31**, 872–888 (2014).

26. Galeote, V. *et al.* Amplification of a Zygosaccharomyces bailii DNA segment in wine yeast genomes by extrachromosomal circular DNA formation. *PloS One* **6**, e17872 (2011).

27. Liti, G., Peruffo, A., James, S. A., Roberts, I. N. & Louis, E. J. Inferences of evolutionary relationships from a population survey of LTR-retrotransposons and telomeric-associated sequences in the Saccharomyces sensu stricto complex. *Yeast Chichester Engl.* **22**, 177–192 (2005).

28. Hou, J., Friedrich, A., Gounot, J.-S. & Schacherer, J. Comprehensive survey of condition-specific reproductive isolation reveals genetic incompatibility in yeast. *Nat. Commun.* **6**, 7214 (2015).

29. Bergman, C. M. Horizontal transfer and proliferation of Tsu4 in Saccharomyces paradoxus. *Mob. DNA* **9**, 18 (2018).

30. Koren, S. *et al.* Canu: scalable and accurate long-read assembly via adaptive k-mer weighting and repeat separation. *Genome Res.* **27**, 722–736 (2017).

31. Vaser, R., Sović, I., Nagarajan, N. & Šikić, M. Fast and accurate de novo genome assembly from long uncorrected reads. *Genome Res.* **27**, 737–746 (2017).

32. Walker, B. J. *et al.* Pilon: an integrated tool for comprehensive microbial variant detection and genome assembly improvement. *PloS One* **9**, e112963 (2014).

33. Kolmogorov, M., Raney, B., Paten, B. & Pham, S. Ragout-a reference-assisted assembly tool for bacterial genomes. *Bioinforma. Oxf. Engl.* **30**, i302-309 (2014).

34. Bonfield, J. K. & Whitwham, A. Gap5--editing the billion fragment sequence assembly. *Bioinforma. Oxf. Engl.* **26**, 1699–1703 (2010).

35. Marçais, G. *et al.* MUMmer4: A fast and versatile genome alignment system. *PLoS Comput. Biol.* **14**, e1005944 (2018).

36. Tao, Y.-T. *et al.* Intraspecific Diversity of Fission Yeast Mitochondrial Genomes. *Genome Biol. Evol.* **11**, 2312–2329 (2019).

37. Coil, D., Jospin, G. & Darling, A. E. A5-miseq: an updated pipeline to assemble microbial genomes from Illumina MiSeq data. *Bioinforma. Oxf. Engl.* **31**, 587–589 (2015).
38. Hunt, M. *et al.* Circlator: automated circularization of genome assemblies using long sequencing reads. *Genome Biol.* **16**, 294 (2015).
39. SAMtoBAM. SAMtoBAM/PhasedDiploidGenomeAssemblyPipeline: v1. (2023) doi:10.5281/zenodo.8068328.
40. Louvel, H., Gillet-Markowska, A., Liti, G. & Fischer, G. A set of genetically diverged Saccharomyces cerevisiae strains with markerless deletions of multiple auxotrophic genes. *Yeast* **31**, 91–101 (2014).
41. Li, H. Aligning sequence reads, clone sequences and assembly contigs with BWA-MEM. Preprint at https://doi.org/10.48550/arXiv.1303.3997 (2013).
42. Danecek, P. *et al.* Twelve years of SAMtools and BCFtools. *GigaScience* **10**, giab008 (2021).
43. DePristo, M. A. *et al.* A framework for variation discovery and genotyping using next-generation DNA sequencing data. *Nat. Genet.* **43**, 491–498 (2011).
44. Danecek, P. *et al.* The variant call format and VCFtools. *Bioinformatics* **27**, 2156–2158 (2011).
45. Li, H. Minimap2: pairwise alignment for nucleotide sequences. *Bioinformatics* **34**, 3094–3100 (2018).
46. Martin, M. *et al.* WhatsHap: fast and accurate read-based phasing. 085050 Preprint at https://doi.org/10.1101/085050 (2016).
47. Abou Saada, O., Tsouris, A., Eberlein, C., Friedrich, A. & Schacherer, J. nPhase: an accurate and contiguous phasing method for polyploids. *Genome Biol.* **22**, 126 (2021).
48. Yue, J.-X. & Liti, G. Long-read sequencing data analysis for yeasts. *Nat. Protoc.* **13**, 1213–1231 (2018).
49. SAMtoBAM. SAMtoBAM/MUMandCo: v3.8. (2023) doi:10.5281/zenodo.8068284.
50. O'Donnell, S. & Fischer, G. MUM&Co: accurate detection of all SV types through whole-genome alignment. *Bioinforma. Oxf. Engl.* **36**, 3242–3243 (2020).

51. Sedlazeck, F. J. *et al.* Accurate detection of complex structural variations using single-molecule sequencing. *Nat. Methods* **15**, 461–468 (2018).
