# Supplementary material for: Telomere-to-telomere assemblies of 142 strains characterize the genome structural landscape in Saccharomyces cerevisiae
Source: Nat Genet. 2023 Jul 31;55(8):1390–9. doi: 10.1038/s41588-023-01459-y (PMC10412453; doi:10.1038/s41588-023-01459-y)
Supplement: Supplementary file 2 — Reporting Summary [file 41588_2023_1459_MOESM2_ESM.pdf]

nature portfolio

Corresponding author(s): Joseph Schacherer, Gianni Liti and Gilles Fischer

Last updated by author(s): 2023 02 14

Reporting Summary

Nature Portfolio wishes to improve the reproducibility of the work that we publish. This form provides structure for consistency and transparency in reporting. For further information on Nature Portfolio policies, see our Editorial Policies and the Editorial Policy Checklist.

Statistics

For all statistical analyses, confirm that the following items are present in the figure legend, table legend, main text, or Methods section.

n/a	Confirmed	
☐	☒	The exact sample size (*n*) for each experimental group/condition, given as a discrete number and unit of measurement
☐	☒	A statement on whether measurements were taken from distinct samples or whether the same sample was measured repeatedly
☐	☒	The statistical test(s) used AND whether they are one- or two-sided<br>*Only common tests should be described solely by name; describe more complex techniques in the Methods section.*
☒	☐	A description of all covariates tested
☒	☐	A description of any assumptions or corrections, such as tests of normality and adjustment for multiple comparisons
☐	☒	A full description of the statistical parameters including central tendency (e.g. means) or other basic estimates (e.g. regression coefficient) AND variation (e.g. standard deviation) or associated estimates of uncertainty (e.g. confidence intervals)
☐	☒	For null hypothesis testing, the test statistic (e.g. *F*, *t*, *r*) with confidence intervals, effect sizes, degrees of freedom and *P* value noted<br>*Give P values as exact values whenever suitable.*
☒	☐	For Bayesian analysis, information on the choice of priors and Markov chain Monte Carlo settings
☒	☐	For hierarchical and complex designs, identification of the appropriate level for tests and full reporting of outcomes
☐	☒	Estimates of effect sizes (e.g. Cohen's *d*, Pearson's *r*), indicating how they were calculated

Our web collection on statistics for biologists contains articles on many of the points above.

Software and code

Policy information about availability of computer code

Data collection	Data collection:<br>Illumina paired end (HiSeq2500) and Oxford Nanopore Technology reads (Minion or Promethion) were generated for 100 strains. All other data was publicly available as indicated in the methods.
Data analysis	All custom scripts and softwares used in the study are publicly available for download:<br>Telofinder: https://github.com/GillesFischerSorbonne/telofinder<br>Script for aneuploidy detction: https://github.com/SAMtoBAM/aneuploidy_detection<br>Script for converting the vcf file into the fasta format: https://github.com/edgardomortiz/vcf2phylip<br>Script for down-sampling paired-end reads with seqtk: https://github.com/lh3/seqtk<br>Script for generating the non-redundant SV dataset: https://github.com/SAMtoBAM/MUMandCo/tree/master/nonredundant_population_datasets<br>Script for haplotype phasing pipeline is available at https://github.com/SAMtoBAM/PhasedDiploidGenomeAssemblyPipeline<br><br>The version number of the software/tools that we used are the following:<br>ADMIXTURE (version: 1.3.0)<br>A5-miseq (version: 20160825)<br>Guppy (version: 3.4.5)<br>Porechop (version: 0.2.4)<br>Picopore (version: 1.2.0)<br>ont-fast5-api (version: 0.3.2)<br>LRSDAY (version: 1.6.0)

Canu (version: 2.0)
SMARTdenovo (version: 5cc1356)
Racon (version: 1.4.7)
Medaka (version: 0.8.1)
Pilon (version: 1.23)
Ragout (version: 2.2)
Filtlong (version: v0.2.0)
Mummer4 (version: 4.0.0beta2)
Samtools (version: 1.11)
Gap5 (version: 1.2.14)
Minimap2 (version: 2.17)
bwa (version: 0.7.17)
GATK3 (version: 3.6-6)
GATK4 (version: 4.1.8.1)
GFF3toolkit (version: 2.1.0)
WhatsHap (version: 1.0)
NGMLR (version: 0.2.7)
Sniffles (version: 2.0.2)
nPhase (version: 1.1.3)
Hapo-G (version: 1)
Seqtk (version: 1.3-r106)
ncbi-BLAST (version: 2.2.31)
RM-BLAST (version: 2.2.28)
Circlator (version: 1.5.5)
MUM&Co (version: 3.8)
paftools (version: 2.17)
bedtools (version: 2.27.1)
Tablet (version: 1.21.0.08)
exonerate(version: 2.2.0)
Maker3 (version: 3.00.0-beta)
EVM (version: 1.1.1)
tRNAscan (version: 1.3.1)
RepeatMasker (version: open-4.0.7)
REannotate (version: 17.03.2015-LongQueryName)
BLAT (version: 36x8)
Proteinortho (version: 5.16b)
Mfannot (version: 1.35)
R (version: 3.5.1)
MACSE (version: 2.04)
IQtree (version: 1.6.12)
vcf2phylip (version: 2.8)
MAFFT (version: 7.471)
Newick Utilities (version: 1.6.0)
ggtree (version: 3.2.1)
phytools (version: 1.0-3)
TreeDist (version: 2.4.1)
MEGA (version: 11.0)
ClipKIT (version: ccc8bf)

For manuscripts utilizing custom algorithms or software that are central to the research but not yet described in published literature, software must be made available to editors and reviewers. We strongly encourage code deposition in a community repository (e.g. GitHub). See the Nature Portfolio guidelines for submitting code & software for further information.

Data

Policy information about availability of data

All manuscripts must include a data availability statement. This statement should provide the following information, where applicable:

- Accession codes, unique identifiers, or web links for publicly available datasets
- A description of any restrictions on data availability
- For clinical datasets or third party data, please ensure that the statement adheres to our policy

All sequencing data and assembly/annotation files were deposited in the European Nucleotide Archive (https://www.ebi.ac.uk/ena/browser/home) under the umbrella project PRJEB59869. The project accession for the raw sequencing data (fast5, nanopore fastq, illumina fastq) is PRJEB50706. The assembly/annotation accessions are PRJEB59413, PRJEB59129, PRJEB59231, PRJEB59232, PRJEB59230 for unphased nuclear, haplotype 1 (HP1), haplotype 2 (HP2), haplotype (HP, for polyploids) and mitochondrial assemblies, respectively. Each accession for individual assemblies is indicated in Supp. Table 1 (nuclear) and Supp. Table 3 (mitochondrial).

Human research participants

Policy information about studies involving human research participants and Sex and Gender in Research.

Reporting on sex and gender	NA
Population characteristics	NA
Recruitment	NA
Ethics oversight	NA

Note that full information on the approval of the study protocol must also be provided in the manuscript.

Field-specific reporting

Please select the one below that is the best fit for your research. If you are not sure, read the appropriate sections before making your selection.

☐ Life sciences ☐ Behavioural & social sciences ☒ Ecological, evolutionary & environmental sciences

For a reference copy of the document with all sections, see nature.com/documents/nr-reporting-summary-flat.pdf

Ecological, evolutionary & environmental sciences study design

All studies must disclose on these points even when the disclosure is negative.

Study description	This study includes 100 newly sequenced genomes (ii) 18 re-assembled genomes and (iii) 24 publically available assemblies. Overall, the study consists of 142 haploid or collapsed assemblies (one per strain), 55 haplotype-resolved assemblies comprising two phased assemblies per heterozygous diploid (21 strains) and one haplo-phased assembly per heterozygous polyploid (13 strains), totaling 197 nuclear genome assemblies. The study also contains 136 mitochondrial chromosome assemblies (114 de novo and 22 publicly available).
Research sample	The study includes 142 Saccharomyces cerevisiae strains.
Sampling strategy	The strains were sampled to represent the species' phylogenetic and ecological diversity, with varying ploidy and heterozygosity levels.
Data collection	Strains were recovered from culture collections.
Timing and spatial scale	NA
Data exclusions	NA
Reproducibility	All genomic assemblies were independently performed twice with 2 different algorithms (Canu and SMARTdenovo)
Randomization	NA
Blinding	Blinding was not relevant because each sequenced strain was unique.

Did the study involve field work? ☐ Yes ☒ No

Reporting for specific materials, systems and methods

We require information from authors about some types of materials, experimental systems and methods used in many studies. Here, indicate whether each material, system or method listed is relevant to your study. If you are not sure if a list item applies to your research, read the appropriate section before selecting a response.

Materials & experimental systems

n/a	Involved in the study
☒	☐ Antibodies
☒	☐ Eukaryotic cell lines
☒	☐ Palaeontology and archaeology
☒	☐ Animals and other organisms
☒	☐ Clinical data
☒	☐ Dual use research of concern

Methods

n/a	Involved in the study
☒	☐ ChIP-seq
☒	☐ Flow cytometry
☒	☐ MRI-based neuroimaging
